# Shared Sanitation versus Individual Household Latrines: A Systematic Review of Health Outcomes

**DOI:** 10.1371/journal.pone.0093300

**Published:** 2014-04-17

**Authors:** Marieke Heijnen, Oliver Cumming, Rachel Peletz, Gabrielle Ka-Seen Chan, Joe Brown, Kelly Baker, Thomas Clasen

**Affiliations:** 1 Faculty of Infectious and Tropical Diseases, London School of Hygiene & Tropical Medicine, London, United Kingdom; 2 Department of Environmental Health, Rollins School of Public Health, Emory University, Atlanta, Georgia, United States of America; 3 School of Civil and Environmental Engineering, Georgia Institute of Technology, Atlanta, Georgia, United States of America; Iran University of Medical Sciences, Iran (Islamic Republic of)

## Abstract

**Background:**

More than 761 million people rely on shared sanitation facilities. These have historically been excluded from international sanitation targets, regardless of the service level, due to concerns about acceptability, hygiene and access. In connection with a proposed change in such policy, we undertook this review to identify and summarize existing evidence that compares health outcomes associated with shared sanitation versus individual household latrines.

**Methods and Findings:**

Shared sanitation included any type of facilities intended for the containment of human faeces and used by more than one household, but excluded public facilities. Health outcomes included diarrhoea, helminth infections, enteric fevers, other faecal-oral diseases, trachoma and adverse maternal or birth outcomes. Studies were included regardless of design, location, language or publication status. Studies were assessed for methodological quality using the STROBE guidelines.

Twenty-two studies conducted in 21 countries met the inclusion criteria. Studies show a pattern of increased risk of adverse health outcomes associated with shared sanitation compared to individual household latrines. A meta-analysis of 12 studies reporting on diarrhoea found increased odds of disease associated with reliance on shared sanitation (odds ratio (OR) 1.44, 95% CI: 1.18–1.76).

**Conclusion:**

Evidence to date does not support a change of existing policy of excluding shared sanitation from the definition of improved sanitation used in international monitoring and targets. However, such evidence is limited, does not adequately address likely confounding, and does not identify potentially important distinctions among types of shared facilities. As reliance on shared sanitation is increasing, further research is necessary to determine the circumstances, if any, under which shared sanitation can offer a safe, appropriate and acceptable alternative to individual household latrines.

## Introduction

Unsanitary disposal of human excreta, together with unsafe drinking water and poor hygiene conditions, is a leading cause of morbidity and mortality in low-income countries [1], [2]. Much of this disease burden consists of diarrhoeal disease, a leading killer of young children. In addition, inadequate sanitation is implicated in schistosomiasis, helminth infections, enteric fevers and trachoma [3]. Lack of access to sanitation also has significant non-health consequences, particularly for women and girls, including lack of security and privacy, decreased school attendance and basic human dignity [4].

An estimated 2.5 billion people lack access to improved sanitation facilities [5]. In developing regions where people are most vulnerable to infection, only one in every three people has access to improved sanitation [5]. At the current pace, the Millennium Development Goal (MDG) sanitation target—to halve the proportion of people with access to basic sanitation by 2015—is set to miss the target by half a billion people [5].

The MDG target, which is expressed in terms of ‘basic sanitation’, was deemed to be context specific and to include ‘the lowest-cost option for securing sustainable access to safe, hygienic, and convenient facilities and services for excreta and sullage disposal that provide privacy and dignity, while at the same time ensuring a clean and healthful living environment both at home and in the neighbourhood of users’ [6]. However, the Joint Monitoring Programme for Water Supply and Sanitation (JMP), which monitors progress toward the target, defines “improved sanitation” in terms of service levels. This includes a private flush or pour-flush toilet or latrine connected to a piped sewer system or septic system, a simple pit latrine with a slab, a ventilated improved pit latrine or a composing toilet. Any other flush or pour-flush latrine, an open pit latrine, bucket latrine, a hanging latrine, or open defecation is “unimproved” and not scored toward the MDG target [5].

Significantly, public and other “shared facilities”—those used by two or more households—are excluded from the definition of “improved sanitation” regardless of the service level [7]. The reason stems from concerns that shared facilities are unacceptable, both in terms of cleanliness (toilets may not be hygienic and fully separate human waste from contact with users) and accessibility (facilities may not be available at night or during periods of high demand) [5].

Nevertheless, shared facilities represent a large and growing proportion of sanitation options available in low-income countries. Nearly a fifth of the population of sub-Saharan Africa (18%) and Eastern Asia (19%) reports using shared sanitation; the practice is particularly common in Ghana (59%), Congo, and Gabon (both 34%) [5]. Globally, the number of users has increased by 437 million since 1990 – increasing from 6 per cent of the global population to 11 per cent in 21 years. In many countries, particularly in crowded urban areas, shared sanitation is the only viable option for those wishing to avoid open defecation; in rural areas, families often keep costs down by sharing latrines between one or more households with family ties [8]. In addition, shared sanitation might provide the opportunity for individuals to move away from open defecation and take the first step on the sanitation ladder.

Perhaps as a result, the JMP is considering a revision to its policy that would include shared sanitation as “improved” – and thus scored toward the post-MDG targets – if the facilities meet the required level of service and are shared among no more than 5 families or 30 persons, whichever is fewer [5]. This proposed change is based on advice from an expert committee [9].

We undertook this review to examine the evidence comparing the impact of shared sanitation versus individual household latrines (IHLs) on health outcomes.

## Methods

The review was undertaken in accordance with a protocol, a copy of which is available on request.

### Eligibility criteria

Studies were eligible for inclusion if they compared health outcomes of populations relying on shared sanitation with those relying on IHLs. In some cases the latrine type was inferred from the study report. For purposes of this review, shared sanitation included any type of facilities intended for the containment of human faeces and used primarily from home; this excludes “public” sanitation facilities designed primarily for use by householders when they are away from the home, such as schools, markets, train or bus stations, city streets, health facilities, governmental buildings or other public places. Health outcomes included diarrhoea, helminth infections, enteric fevers, other faecal-oral diseases, trachoma and adverse maternal or birth outcomes. Studies were included regardless of study design, location, language or publication status.

### Information sources

Our search was performed through September 2013. We employed keywords for health related outcomes. The full lists of key search terms are listed in Table S2.

We performed an electronic search of 19 databases, including 2 Chinese language databases. An overview of the databases is shown in Table 1. Where possible, the same key search terms were used to search the grey literature sources for relevant literature. Conference proceedings from the following institutions were searched for relevant abstracts: WEDC (Loughborough University), IRC International Water and Sanitation Centre, and the German Agency for International Cooperation (GIZ). In addition, governmental agencies, non-governmental organisations (NGOs), universities and others involved in funding, implementing or investigating sanitation were contacted to solicit other studies that met the review's inclusion criteria. In all cases, references lists of studies were also reviewed for additional possible studies.

**Table 1:** Electronic databases searched.

Database		Last search date	Number of results
OvidSP (Ovid Technologies 2013)	EMBASE	October 7^th^, 2013	4248
	MEDLINE	October 7^th^, 2013	2976
	CAB Abstracts,	October 12^th^, 2013	6586
	Global Health,	October 7^th^, 2013	5660
	HMIC,	October 7^th^, 2013	74
	Social Policy & Practice	October 7^th^, 2013	42
Virtual Health Library	DESASTRES	October 3^rd^, 2013	332
	LEYES	October 3^rd^, 2013	29
	LILACS	October 3^rd^, 2013	36
	MedCarib	October 3^rd^, 2013	28
	REPIDISCA	October 3^rd^, 2013	73
Individually searched databases	Africa wide	October 4^th^, 2013	3495
	Cochrane	October 3^rd^, 2013	16
	IMEMR	October 4^th^ 2013	10
	CEHA	October 4^th^, 2013	2
	HISA	October 4^th^, 2013	5
	WPRIM	October 4^th^, 2013	4
Chinese language databases	WANFANG	October 23^rd^, 2013	915
	CNKI	Ocotber 23^rd^, 2013	946

### Study selection

Two authors independently examined the full text of potentially relevant articles using the standard protocol developed by the authors. For Chinese-language search results, a third author undertook the same process individually.

### Data collection process

Relevant data, including a brief description of the study (study design, setting and year), details of the study population, specifications of the sanitation facilities and the outcome measures investigated were extracted independently from all eligible studies by two authors. If an article lacked necessary information, we contacted the authors or publishers to attempt to secure it.

### Assessment for methodological quality

Each study included in the review was assessed for methodological quality. For observational studies, the STROBE (Strengthening of the Reporting of Observational studies in Epidemiology) statement was used as a guideline to extract data on the risks of bias. While the protocol for the review contemplated assessing studies with a specified intervention group using the Cochrane EPOC (Effective Practice and Organisation of Care) criteria, no such studies met the review's inclusion criteria.

### Synthesis of results

We pooled studies reporting on diarrhoea and conducted a meta-analysis based on a random effects model. No further synthesis of results was undertaken to due to the limited number of studies reporting on other health outcomes.

## Results

### Study selection

**Figure 1:**
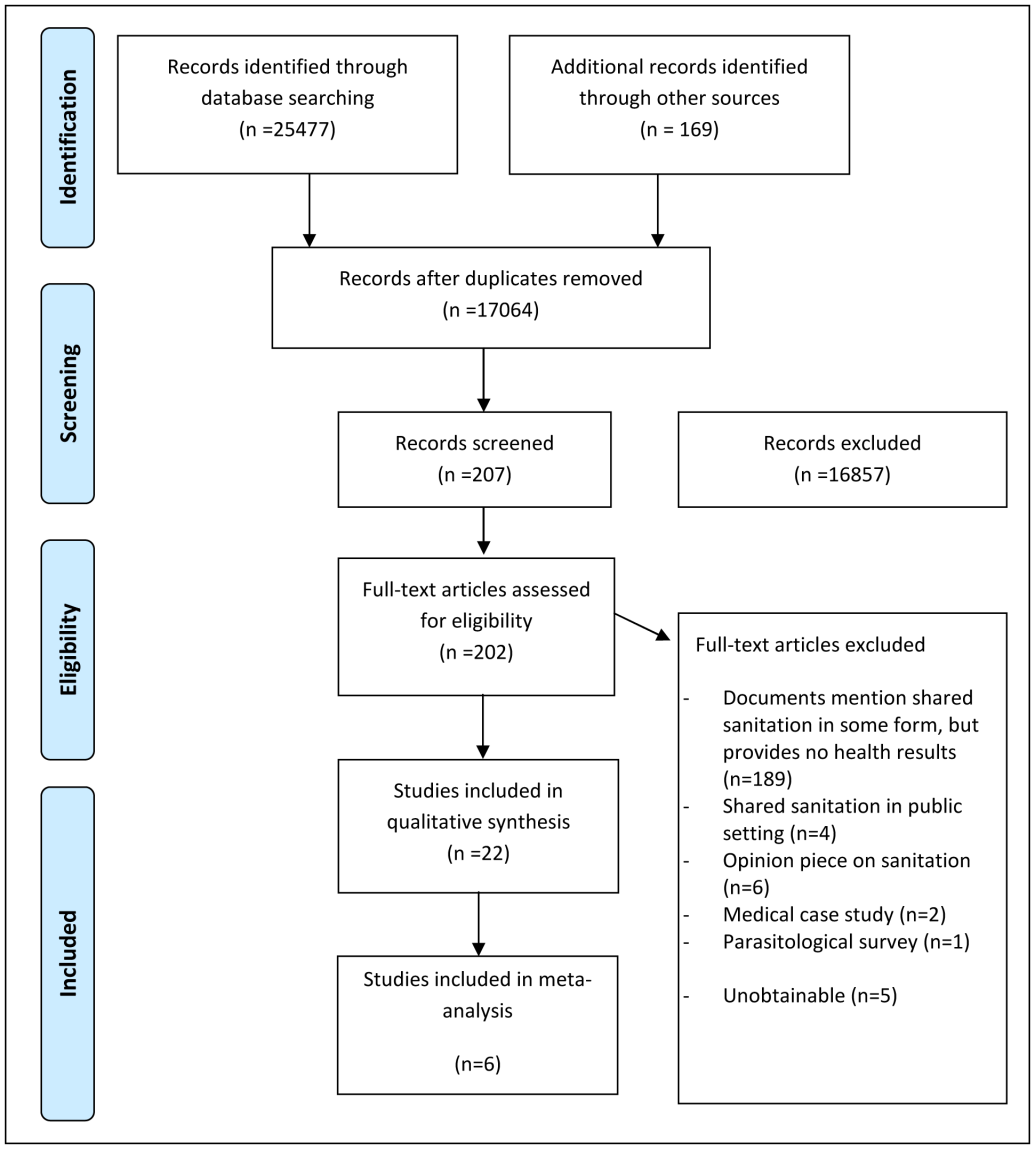
PRIMSA Flow Chart.

Execution of the search strategy resulted in 25477 titles and abstracts. In addition, 169 unpublished documents were retrieved. These titles and abstracts were screened and the full text articles of 202 documents were obtained for further assessment. Of these studies, 22 documents met the review's inclusion criteria. A detailed overview is provided in Figure 1. Reasons for exclusion of documents are provided in Table S5.

### Study characteristics

General diarrhoea was the outcome of interest in six studies [10]–[15], with two studies focusing specifically on watery diarrhoea [16], [17] and another on bloody diarrhoea [18]. While other studies included all ages, Baker et al. [14], Chakraborty et al. [13], and Sobel et al. [12] limited the studies to diarrhoea in children under the age of 5 years. A variety of intestinal parasites were investigated in seven studies [11], [19]–[24]. Other health outcome measures included *S.typhi* and *S. paratyphi* A [25], poliomyelitis [26], trachoma [27], *Shigella dysenteriae* type I [28], perinatal death and antenatal foetal death [29], preterm birth and low birth weight [30], and hospital admissions [31]. One study investigated diarrhoea specifically in an HIV-positive population [15].

#### Participants and settings

Most studies took place in urban settings, though one conducted a comparative urban-rural investigation [23]. Except for one study among an aboriginal population in Australia [31], all studies were conducted in low- and middle- income settings. Three studies were conducted in Kenya [16]–[18] and two in India [10], [13], Bangladesh [11], [21], and in Egypt [20], [22]; and one in each of Brazil [12], Zambia [28], the Democratic Republic of Congo [24], Nigeria [30], Malawi [23], Zimbabwe [19], Taiwan [26], Jamaica [29], Ghana [32], Nepal [25], South Africa [15] and Tanzania [27]. One study was conducted in multiple countries [14]. Two studies, Shultz et al. and Mahamud et al., were conducted in long-established refugee camps [16], [17].

The study population varied considerably, from only women in the studies on maternal and new born health [29], [30], to only men in a study in Egypt [20]. Seven studies focused specifically on children, with ages ranging from children under the age of 5 [13], [14], [22], children under the age of three [10], children aged 1–5 [12], [27] and children aged 3–14 years old [23]. As many of the health outcomes vary considerably with age, socio-economic class, population density and other covariates, the comparability of these results must be viewed with the significant differences in study populations and settings in mind.

#### Types of shared sanitation

The types of latrines assessed and reported on also varied considerably (Table 2). In most cases, the common facilities were shared with other families [14], [18], [23], [31]; only Montgomery et al. provided information on the number of families sharing [27]. Shultz et al. looked at three or more households sharing a latrine (without a clearly specified comparison group) [16]. In some instances IHL was compared to ‘sharing with at least one other family’ [12], [22], [26]. Olusanya et al. [30] compared shared latrines with IHLs, though with no further details of the type of shared latrine. Similarly, Karkey et al. compared household latrines use versus community latrines [25], whereas Moshabela et al. report sharing sanitation facilities with an average of two other households [15].

**Table 2:** Summary of data extracted from included studies.

Author	Study design	Type of Shared Sanitation	Type of Comparison Sanitation	Main outcomes	Summary measures
Brooks 2003	Case control	‘allowing other families to use the compound latrine’	Latrine for private use only	Risk factors for bloody diarrhoea	Matched Odds Ratio (95% CI)
Chakraborty 1983	Cross sectional	Community latrines in slum	Private latrine connected to sewer	Episodes of diarrhoea	Mean
Chandiwana 1989	Cross sectional	Shared latrines	No comparison	Prevalence and intensity of hookworm and roundworm	Prevalence, correlations
Curtale 1998	Cross sectional	Family latrine not shared with others	Latrine shared with others	Prevalence and intensity of intestinal helminth infection	Prevalence
Ghosh 1994	Case control	Sharing latrine	Private latrine*	Diarrhoeal disease	Percentages
Golding 1994	Cross sectional	Toilet used by others outside of family	Toilet only used by family	Perinatal death, antepartum fetal death	Adjusted OR (95% CI)
Hall 1994	Cross sectional	Shared and community latrine	Private latrine	*Strongyloides stercoralis* infection	Odds ratio (95% CI)
Khan 1987	Cross sectional	Communal latrines in peri urban slums	Open pit latrines in peri urban slums	Diarrhoea cases and intestinal parasite prevalence†	Prevalence
Kim-Farley 1984	Case control	Toilet shared >1 family	Private latrines*	Poliomyelitis	Odds ratio (95% CI)
Mahfouz 1997	Cross sectional	Sharing toilets with other family	Sole use of household latrine*	Prevalence of intestinal parasites and protozoa	Adjusted OR (95% CI)
Montgomery 2010	Case control	Shared latrines	Private latrines	Trachoma	Adjusted OR (95% CI)
Munoz 1992	Cohort	Communal toilet	Private toilet	Hospital admissions	Percentages, factor scores
Olusanya 2010	Cross sectional	Shared sanitation	Private sanitation	Preterm and low birthweight	Unadjusted OR (95% CI)
Phiri 2001	Cross sectional	Shared latrine	Private latrine*	Prevalence of helminths	Adjusted OR (95% CI)
Shultz 2009	Case control	Three or more households sharing same latrine	Not specified	Watery diarrhoea	Matched OR (95% CI)
Sobel 2004	Case control	Shared latrine with other household	Private latrine*	Acute diarrhoeal disease	Matched OR (95% CI)
Tshikuka 1994	Cross sectional	Sharing a toilet with others	Private latrine*	*Ascaris lumbricoides* infection	Means, Beta coefficient
Tuttle 1995	Case control	Shared latrine	Private latrine*	*Shigella dysenteriae* type1	Matched OR (95% CI)
Baker 2011	Case control (abstract)	Shared sanitation	Private latrine	Risk of diarrhoea	Matched OR (95% CI)
Moshabela 2012	Case control	Sharing latrine with an average of 2 households	Private latrine*	Diarrhoeal disease	Prevalence
Karkey, 2013	C ase control	Community latrine	Household latrine	Enteric infection (*S. typhi* or *S. paratyphi* A.)	Adjusted OR (95% CI)
Mahamud 2012	Case control	Communal latrine	Compound latrine	Diarrhoea and Cholera	Odds ratio (95% CI)

*Latrine type inferred from study report.

† Study mentions measurement of incidence. As this is a cross sectional study, it is interpreted as prevalence.

In several cases, the type of shared sanitation was not well defined, with the authors using terms such as “communal” [13], [21] to distinguish them from IHLs. Moreover, potentially important information such as ownership, management or approximate numbers of users was often omitted.

Ghosh [10] and Tuttle [28] looked at the sharing of a common latrine, and Golding [29] considered the sharing of toilets; in these cases, however, it was not clear that the comparison was an IHL. Tshikuka et al. investigated the number of persons per toilet as well as the number of people practicing open defecation [24]. Chandiwana et al. looked at the number of persons per latrine, without specifying a comparison group [19]. In these two cases where the number of people per toilet was reported, it was not clear whether this was actually counted, or if an average of households or persons per latrine was calculated.

Some studies included multiple comparisons, for example, Khan reported on communal latrines versus private or compound shared latrines [11] whereas Curtale looked at different settings, including rural IHLs and some sharing of family latrines in urban areas [20].

#### Study designs

All studies included in the review followed an observational study design. These were either cross-sectional, case control or cohort studies (Table 2).

#### Summary measures

The large variety of studies included resulted in different study measures (Table 2). Odds ratios were reported in 14 studies and for the remainder of studies only the percentages or differences in means were reported.

### Assessment of methodological quality

The Supplementary Material provides detailed information on the methodological assessments (Table S3). Only one of the included studies reported a sample size calculation [23]. Similarly, only one study reported the interview response rate [27]. Seven studies reported using some form of random sampling [13], [15], [17], [20], [23]–[25], though only four of these clearly described the randomisation method [15], [17], [23], [25]. Eight of the 11 included case control studies report matching of the cases and controls (matched [12], [14], [16]–[18], [25], [27], [28], while three used unmatched cases and controls [10], [15], [26].)

Among the nine studies reporting on diarrhoea, only Baker et al. and Shultz et al. used clinically confirmed cases. All others relied on self-reported diarrhoea and failed to report on the recall period, both potential sources of bias [33].

### Outcomes

Twenty-two studies reported on health outcomes associated with shared sanitation. These are summarized in Table 3.

**Table 3:** Summary of health outcomes.

Author	Study design	Main outcomes	Outcome measure	
**Diarrhoea**				
Brooks 2003	Case control	Risk factors for bloody diarrhoea	OR 2.40 (95% CI 1.19–4.48)	
Chakraborty 1983	Cross sectional	Episodes of diarrhoea	On average, there were 1.6 episodes of diarrhoea in the slum, versus 1.4 in the housing project	
Khan 1987	Cross sectional	Diarrhoea cases and intestinal parasite prevalence	On average, there were 0.81 episodes of diarrhoea in the area with communal latrines, versus 0.7 in the area with open pit latrines (p<0.01). No CI.	
Baker 2011	Case control (abstract)	Risk of severe to moderate diarrhoea	OR 1.20 (95% CI 1.1–1.3)	
Shultz 2009	Case control	Watery diarrhoea	OR 2.17 (95% CI 1.01–4.68)	
Sobel 2004	Case control	Acute diarrhoeal disease	OR 1.48 (95% CI 1.07–2.04)	
Ghosh 1994	Case control	Diarrhoeal disease	P = 0.008	No CI.
Moshabela 2012	Case control	Diarrhoeal disease	25.3% of cases and 23.7% of controls (p = 0.76)reported sharing sanitation	
Mahamud 2012	Case control	Watery diarrhoea/cholera	OR 3.33 (95% CI 1.34–8.30)	
**Helminths**				
Chandiwana 1989	Cross sectional	Prevalence and intensity of hookworm and roundworm	Correlations between number of households and hookworm r = 0.72, (P<0.1), roundworm r = −0.009, (P<0.1)	
Curtale 1998	Cross sectional	Prevalence and intensity of intestinal helminth infection	Sharing latrines and the absence of piped water in the house were associated with a significantly higher intensity of infection for *A. Lumbricoides* (p<0.001) and *T. Trichiura* (p<0.05)	
Hall 1994	Cross sectional	*Strongyloides stercoralis* infection	OR 2.72 (95% CI 1.57–4.72)	
Mahfouz 1997	Cross sectional	Prevalence of intestinal parasites and protozoa	Intestinal helminths: OR 1.95 (95% CI 1.38–2.75) Protozoa: OR 1.65 (95% CI 1.06–2.58)	
Phiri 2001	Cross sectional	Prevalence of helminths		
Tshikuka 1994	Cross sectional	*Ascaris lumbricoides* infection	Nr of persons/toilet Beta 0.45 (P<0.01, SE 0.02)	
**Other health outcomes**				
Tuttle 1995	Case control	*Shigella dysenteriae* type1	OR 3.3 (95% CI 1.1–10.2)	
Karkey 2013	Case control	*S. typhi* and *S. paratyphi A*	aOR 4.92 (1.2–19.5) for *S. paratyphi* A aOR 7.26 (1.4–37.2) for *S.typhi*	
Montgomery 2010	Case control	Trachoma	OR 0.95 (95% CI 0.55–1.67)	
Munoz 1992	Cohort	Hospital admissions	‘communal sanitation’ was a significant variable in the factor analysis (p<0.01)	
Olusanya 2010	Cross sectional	Preterm and low birthweight	Prematurity aOR 1.36 (95% CI 1.07–1.48) Low birth weight aOR 1.27 (95% CI 0.98–1.65)	
Kim-Farley 1984	Case control	Poliomyelitis	OR 4.0 (95% CI 1.9–8.3)	
Golding 1994	Cross sectional	Perinatal death, antepartum fetal death	Antepartum fetal death aOR 1.62 (95% CI 1.28–2.03) Perinatal death aOR 1.41 (95% CI 1.21–1.64)	

#### Diarrhoeal disease

Nine studies investigated diarrhoeal disease as an outcome measure (Table 3). In all but two [13], [15], sharing a latrine was found to be associated with an increased risk of diarrhoeal disease. Shultz et al. found that sharing a latrine with at least three households was associated with an increased risk of watery diarrhoea (Matched OR 2.17 [95% CI 1.01–4.68] [16]. Sobel et al. found that sharing a toilet with another household was a risk factor for acute diarrhoea cases presented at hospital (OR 1.48 [95% CI 1.07–2.04]) [12]. Similarly Tuttle et al. reported that households with shigella cases were more likely to share latrines than control households (Matched OR 3.3 [95% CI 1.1–10.2]) [28]. Initial results from a multi-country study by Baker et al. showed increased odds of moderate and severe diarrhoea when latrines are shared (matched OR 1.2 [95% CI 1.1–1.3]) [14]. A significant association between shared latrines and the incidence of diarrhoea is also reported by Ghosh et al. (p = 0.008) though no odds ratios or confidence intervals are presented [10]. Brooks et al. report an increased risk of bloody diarrhoea if other families are allowed to use the latrine (OR 2.40 [95% CI 1.19–4.48]), though no data is provided on the number of families sharing latrines [18].

Chakraborty et al. found no difference in the incidence of diarrhoea between children living in the slum, where public latrines are available, and children living in a housing project, where each family had their own latrine [13]. It must be noted however, that the study population is children under 5 years, and the study also reports that few of these young children use latrines, irrespective of where they live. Similarly, Moshabela et al. found no difference between diarrhoeal disease for those reporting sharing sanitation facilities with other households (25.3% of cases and 23.7% of controls shared sanitation facilities, p = 0.76). All the subjects in this study were HIV positive individuals [15].

**Figure 2:**
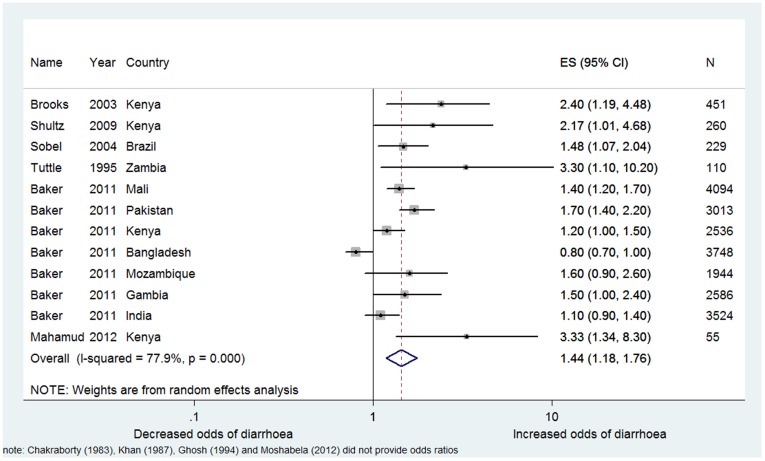
Meta-analysis for the use of shared sanitation and diarrhoea. Image produced using Stata (Statacorp LP, TX USA). CI: Confidence Interval. ES: Effect size (Odds Ratio).

The studies reporting an effect on diarrhoea have been pooled in a meta-analysis using a random effects model (Figure 2). This yielded a pooled odds ratio (OR) of 1.44 (95%CI: 1.18–1.76), suggesting increased risk associated with shared sanitation. The pooled estimate is characterized by substantial heterogeneity (I^2^ = 77.9%). Some of the studies contributing data to our pooled analysis (Figure 2) on the effect of shared sanitation on diarrhoea is drawn from preliminary, yet-to-be published results from seven countries included in the Global Enterics Multi-Centre Study (GEMS) [14]. However, the GEMS study design as well as the general methods for collection of water and sanitation exposure data and the definition for moderate and severe diarrhoea used to screen and enrol case and controls has been published [34], [35]. The study used a case-control design where cases were based on clinical diagnoses of moderate to severe diarrhoea in children <5 years. As shown in Figure 2, initial results, adjusting for wealth and faeces visible in the facility, show that shared sanitation was a statistically significant risk factor in two countries (Pakistan, Mali) and was trending toward increased risk in three other countries (Gambia, Mozambique and Kenya). Interestingly, shared sanitation trended in the opposite direction, appearing protective in Bangladesh. This study reports that even though there is site variability, there is an overall trend among most sites. Except for Bangladesh, cases are more likely to live in a household that shares a latrine. The pooled odds ratio from the seven GEMS studies yields an OR of 1.26 (95% CI 1.01–1.57) compared to OR 2.01 (95% CI 1.44–2.81) for the five published studies (Figure S2).

#### Helminths and protozoan parasites

Six studies reported associations between shared sanitation facilities and helminth infections, of which only one study reported no association (Table 3). Tshikuka et al. found that the number of persons per toilet was statistically associated with *Ascaris lumbricoides* infection intensity [24]. However, it is not clear whether the persons per latrine were counted or calculated as an average. Mahfouz et al. found that sharing toilets with another family increased the risk of intestinal helminths (*A.lumbricoides*, *Trichuris trichiura*, *Hymenolepis nana*, *Oxyuris*, *Ancylostoma duodenale*, *Schistosoma mansoni)* (adjusted OR 1.95[95% CI 1.38–2.75]) and from protozoan parasites [*Giardia lamblia*, *Entamoeba histolytica*] (adjusted OR 1.65 [95% CI 1.06–2.58]) [22].

Hall et al. found that for adults, using a community latrine rather than a private latrine was statistically significant risk factor for *S. stercoralis* infection (adjusted OR 2.72 [95% CI 1.57–4.72) [21]. On the other hand, they found that using a latrine shared between neighbours versus a private latrine showed no significant association. Similarly, for children the risk of *S. stercoralis* infection was increased when using communal latrines (adjusted OR 2.43 [95% CI 1.35–4.38]), whereas no such association could be found for shared latrines. No information was provided on the number of people or households using either the shared or the communal latrines.

In a study in Egypt, sharing latrine with other families and the absence of piped water inside the house were associated with a significantly higher intensity of infection for *A. lumbricoides* (p<0.001) and for *T. trichiura* (p<0.05) but not *for S. mansoni* [20]. No separate data were presented for shared latrines and no information was provided on the number of households sharing. Lastly, Phiri et al. found no statistically significant risk associated with *A. lumbricoides*, hookworm, *T. Trichiura*, or *S. stercoralis* infection and shared latrine facilities [23].

#### Other health outcomes

A study by Montgomery et al. found that shared latrines provided as much protection as private latrines in regard to the risk of trachoma (adjusted OR 0.95 [95% CI 0.55–1.67]) [27]. Also, the number of households sharing did not significantly alter the risk.

Kim-Farley et al. investigated a poliomyelitis outbreak in Taiwan using a case control design [26]. It was shown that more cases than controls shared toilets with other families (OR 4.0 [95% CI 1.9–8.3]). However, this was a univariate analysis, not controlled for other exposures.

Karkey et al. investigate enteric infection with either *S. typhi* or *S. paratyphi* A and found that communal latrine use (versus individual household latrines) was protective (adjusted OR 4.92 [95% CI 1.2–19.5] for *S. paratyph*i A and adjusted OR 7.26 [95% CI 1.4–37.2] for *S. typhi*). In this study, 92.2 per cent of the cases used a household latrine versus 77.9 per cent of the controls [25].

Several studies reported on adverse birth outcomes. Olusanya et al. investigated preterm birth and low birth weight risk factors [30]. Living in a house with shared sanitation facilities was found to be a risk factor for prematurity (adjusted OR 1.26 [95% CI 1.07–1.48]), whereas there was only a weak association with low birth weight (adjusted OR 1.27 [95% CI 0.98–1.65]). Golding and colleagues found an increased risk of perinatal death among women who had to share toilet facilities with people other than members of their family [29]. This was associated especially with antepartum fetal deaths (adjusted OR 1.62 [95% CI 1.28–2.03]) and perinatal death (adjusted OR 1.41 [95% CI 1.21–1.64]). In rural aboriginal communities in Australia, Munoz et al. reported that communal toilets were associated with an increased risk of hospital admissions for children [31]. However, the authors acknowledged that many community characteristics were strongly associated with differences in admission rates between communities thus limiting the potential for causal interferences.

## Discussion

In general, the evidence suggests that those relying on shared sanitation facilities compared to IHLs are at increased risk of adverse health outcomes, including diarrhoeal disease, helminth infection, poliomyelitis, as well as prematurity, antepartum fetal death and perinatal death. The evidence on diarrhoeal disease and on helminth infection reflects a consistent pattern across most studies and study sites, while the evidence on poliomyelitis and adverse birth outcomes was generated from single studies. On the other hand, research found no increased risk of trachoma associated with reliance on shared sanitation.

Although most of the studies reviewed suggest a pattern of shared sanitation and adverse health outcomes, the quality of these studies varies and the actual strength of evidence is weak, and should be interpreted with caution. This is due to at least four major limitations.

First, as noted, many of the studies included in the review are of uncertain methodological quality. Fewer than a third of the studies reported using random selection of the study sites or population, presenting the potential for selection bias. The type of sanitation facilities being compared was not blinded to the study population or assessors. This and the fact that many studies relied on reported outcomes raises questions of reporting bias. Many of the studies fail to report on case definitions, participant eligibility and selection procedures, methods for assessing outcomes, potential sources of bias, etc. There are also statistical shortcomings, such as the failure to adjust for clustering and the treatment of populations as multiple rather than single units. Moreover, many studies reflect methodological problems common in assessments of environmental health interventions [36] and in the assessment of faecal-oral diseases such as diarrhoea [37].

Second, few of the studies report on possible factors other than the type of sanitation facilities that could be important confounders or effect modifiers. Most obvious of these, perhaps, is actual latrine use. There is evidence, for example, that a variety of factors such a distance, waiting time and cost can significantly impact the use of shared sanitation facilities [38], [39]. Other factors that may vary between shared sanitation facilities and IHLs include latrine maintenance, distance to and quantity/quality of water supplies, the presence and use of hand washing facilities and soap, the manner in which users dispose of child faeces, and the way in which the waste is subsequently removed from the facilities and ultimately disposed of in the setting. Additionally, the population density, socio-economic status, gender or other equity issues of the users of shared facilities versus IHL may differ, aspects which are infrequently reported on in the studies specifying shared sanitation.

Third, there are substantial differences among the studies that limit their comparability. This includes differences in study design, settings, study populations and ambient conditions. It also includes fundamental differences (and in many cases, uncertainty) in the actual types of shared sanitation and the types of IHLs being compared. There are also important differences in outcomes, the manner in which they were assessed and in the methods for their analysis.

Finally, and perhaps most important, the studies undertaken to date allow only for only a weak causal inference between shared sanitation and adverse health outcomes. None of the studies identified in the review followed an experimental design. While many studies adjusted for known confounders, others did not. As observational studies, all are at risk of unknown confounders. We cannot rule out the possibility that that reliance on shared sanitation is simply a proxy for more direct causes of adverse health outcomes.

There is a need for rigorous studies in multiple study settings in order to determine the extent to which reliance on shared sanitation is causally associated with adverse health outcomes. There is also a need to identify the factors that may mitigate or otherwise modify any increased health risk associated with shared sanitation. Studies have found evidence that shared sanitation may be more poorly maintained, more costly, less accessible and less frequently used than IHLs [38]–[41]. These and other factors are likely to vary considerably depending on population density, the ratio of latrines per household or person, the quality of construction and upkeep, and the manner in which the latrines are managed. Future research, using both qualitative and quantitative methods, may help identify the circumstances in which shared sanitation might be a safe and effective alternative for increasing populations that do not have access to IHLs or where household-levels sanitation solutions are not possible or appropriate. Pending this research, policymakers and public health professionals should exercise caution in taking steps that may encourage the promotion of shared sanitation.

## Supporting Information

Checklist S1 (DOCX)

Figure S1 **Sub group forest plot-Published data only.** Image produced using Stata (Statacorp LP, TX USA). CI: Confidence Interval. ES: Effect size (Odds Ratio).
(TIF)

Figure S2 **Sub group forest plot-Unpublished data only.** Image produced using Stata (Statacorp LP, TX USA). CI: Confidence Interval. ES: Effect size (Odds Ratio).
(TIF)

Protocol S1 (PDF)

Table S1 **Key search terms.**
(DOCX)

Table S2 **Search strategy as performed in OVID databases.**
(DOCX)

Table S3 **Methodological quality.**
(DOCX)

Table S4 **Extracted data.**
(DOCX)

Table S5 **Excluded documents.**
(DOCX)

## References

[1] Prüss-Üstün A, Bos R, Gore F, Bartram J (2008) Safer water, Better health- Costs, benefits and sustainability of interventions to protect and promote health. Geneva.

[2] Lim SS, Vos T, Flaxman AD, Danaei G, Shibuya K, et al (2012) A comparative risk assessment of burden of disease and injury attributable to 67 risk factors and risk factor clusters in 21 regions, 1990–2010: a systematic analysis for the Global Burden of Disease Study 2010. Lancet 380: 2224–2260. 23245609 10.1016/S0140-6736(12)61766-8 PMC4156511

[3] Bartram J, Cairncross S (2010) Hygiene, Sanitation, and Water: Forgotten Foundations of Health. PLoS Medicine 7: 11. 10.1371/journal.pmed.1000367 PMC2976722 21085694

[4] Amnesty International (2010) Kenya: Insecurity and indignity: Women's experience in the slums of Nairobi, Kenya.

[5] Joint Monitoring Programme (2013) Progress on Drinking Water and Sanitation: 2013 Update. WHO/UNICEF.

[6] United Nations Millennium Project (2005) Health, Dignity, and Development. What Will it Take? Task Force on Water and Sanitation.

[7] Joint Monitoring Programme (2010) Progress on Sanitation and Drinking Water: 2010 Update.

[8] Joint Monitoring Programme (2012) Progress on Drinking Water and Sanitation: 2012 Update. UNICEF/WHO.

[9] Joint Monitoring Programme (2012) Proposal for consolidated drinking water, sanitation and hygiene targets, indicators and definitions. the Hague, the Netherlands. 3–5 December.

[10] Ghosh S, Sengupta PG, Mandal SK, Manna B, Sikder SN, et al (1994) Maternal behaviour and feeding practices as determinants of childhood diarrhoea: some observations amongst rural Bengalee mothers. Indian Journal of Public Health 38: 2 77–80. 7836002

[11] Khan MU (1987) Limitation of communal latrines in changing the prevalence of parasites and diarrhoeal attack rate in Dhaka Peri-urban slums. Environmental Pollution 47: 3 187–194. 10.1016/0269-7491(87)90209-0 15092706

[12] Sobel J, Gomes TA, Ramos RT, Hoekstra M, Rodrigue D, et al (2004) Pathogen-Specific Risk Factors and Protective Factors for Acute Diarrheal Illness in Children Aged 12–59 Months in Sao Paulo, Brazil. Clinical Infectious Diseases 38: 1545–1551. 15156440 10.1086/420822

[13] Chakraborty A, Das J (1983) Comparative study of incidence of diarrhea among children in two different environmental situations in Calcutta. Indian Pediatrics 20: 12 907–913. 6676302

[14] Baker K, O'Reilly CE, Mintz ED, Farag T, Nasrin D, et al.. The risk of moderate and severe diarrhea in children less than 5 years old is increased among families who share a sanitation facility; Conference proceedings- American Society Tropical Medicine Hygiene; 2011.

[15] Moshabela M, MacPherson P, Ezard N, Frean E, Mashimbye L, et al (2012) Clinical and social determinants of diarrhoeal disease in a rural HIV/AIDS clinic, South Africa: A case-control study. International Journal of STD and AIDS 23: 5 346–350. 22648889 10.1258/ijsa.2011.011285 PMC3966081

[16] Shultz A, Omollo JO, Burke H, Qassim M, Ochieng JB, et al (2009) Cholera outbreak in Kenyan refugee camp: risk factors for illness and importance of sanitation. American Journal of Tropical Medicine & Hygiene 80: 4 640–645. 19346392

[17] Mahamud AS, Ahmed JA, Nyoka R, Auko E, Kahi V, et al (2012) Epidemic cholera in Kakuma Refugee Camp, Kenya, 2009: The importance of sanitation and soap. Journal of Infection in Developing Countries 6: 3 234–241. 10.3855/jidc.1966 22421604

[18] Brooks JT, Shapiro RL, Kumar L, Wells JG, Phillips-Howard PA, et al (2003) Epidemiology of sporadic bloody diarrhea in rural Western Kenya. American Journal of Tropical Medicine and Hygiene 68: 6 671–677. 12892051

[19] Chandiwana SK, Bradley M, Chombo F (1989) Hookworm and roundworm infections in farm-worker communities in the large-scale agricultural sector in Zimbabwe. Journal of Tropical Medicine and Hygiene 92: 5 338–344. 2810452

[20] Curtale F, Shamy MY, Zaki A, Abdel-Fattah M, Rocchi G (1998) Different patterns of intestinal helminth infection among young workers in urban and rural areas of Alexandria Governorate, Egypt. Parassitologia 40: 3 251–254. 10376279

[21] Hall A, Conway DJ, Anwar KS, Rahman ML (1994) Strongyloides stercoralis in an urban slum community in Bangladesh: factors independently associated with infection. Transactions of the Royal Society of Tropical Medicine and Hygiene 88: 5 527–530. 10.1016/0035-9203(94)90146-5 7992327

[22] Mahfouz AAR, El-Morshedy H, Farghaly A, Khalil A (1997) Ecological determinants of intestinal parasitic infections among pre-school children in an urban squatter settlement of Egypt. Journal of Tropical Pediatrics 43: 6 341–344. 10.1093/tropej/43.6.341 9476455

[23] Phiri K, Whitty CJM, Graham SM, SSembatya Lule G (2000) Urban/rural differences in prevalence and risk factors for intestinal helminth infection in southern Malawi. Annals of Tropical Medicine and Parasitology 94: 4 381–387. 10.1080/00034983.2000.11813553 10945048

[24] Tshikuka JG, Scott ME, Gray-Donald K (1995) Ascaris lumbricoides infection and environmental risk factors in an urban African setting. Annals of Tropical Medicine and Parasitology 89: 5 505–514. 10.1080/00034983.1995.11812983 7495364

[25] Karkey A, Thompson CN, Tran Vu Thieu N, Dongol S, Le Thi Phuong T, et al (2013) Differential Epidemiology of Salmonella Typhi and Paratyphi A in Kathmandu, Nepal: A Matched Case Control Investigation in a Highly Endemic Enteric Fever Setting. PLoS Neglected Tropical Diseases 7: 8. 10.1371/journal.pntd.0002391 PMC3749961 23991240

[26] Kim-Farley RJ, Rutherford G, Lichfield P, Hsu ST, Orenstein WA, et al (1984) Outbreak of paralytic poliomyelitis, Taiwan. Lancet 2: 8415 1322–1324. 10.1016/s0140-6736(84)90831-6 6150332

[27] Montgomery MA, Desai MM, Elimelech M (2010) Comparing the effectiveness of shared versus private latrines in preventing trachoma in rural Tanzania. American Journal of Tropical Medicine and Hygiene 82: 4 693–695. 20348521 10.4269/ajtmh.2010.09-0540 PMC2844581

[28] Tuttle J, Ries AA, Chimba RM, Perera CU, Bean NH, et al (1995) Antimicrobial-resistant epidemic Shigella dysenteriae type 1 in Zambia: modes of transmission. The Journal of infectious diseases 171: 2 371–375. 10.1093/infdis/171.2.371 7844374

[29] Golding J, Greenwood R, McCaw-Binns A, Thomas P (1994) Associations between social and environmental factors and perinatal mortality in Jamaica. Paediatric and Perinatal Epidemiology 8: Suppl 1 17–39. 10.1111/j.1365-3016.1994.tb00489.x 8072899

[30] Olusanya BO, Ofovwe GE (2010) Predictors of preterm births and low birthweight in an inner-city hospital in sub-Saharan Africa. Maternal and Child Health Journal 14: 6 978–986. 10.1007/s10995-009-0528-4 19795198

[31] Munoz E, Powers JR, Nienhuys TG, Mathews JD (1992) Social and environmental factors in 10 Aboriginal communities in the Northern Territory: Relationship to hospital admissions of children. Medical Journal of Australia 156: 8 529–533. 10.5694/j.1326-5377.1992.tb121412.x 1565044

[32] Fobil J, May J, Kraemer A (2010) Assessing the relationship between socioeconomic conditions and urban environmental quality in Accra, Ghana. International Journal of Environmental Research and Public Health. 10.3390/ijerph7010125 PMC2819780 20195437

[33] Schmidt WP, Boisson S, Genser B, Baretto ML, Baisely K, et al (2010) Weight-for-age z-score as a proxy marker for diarrhoea in epidemiological studies. Journal of Epidemiology and Community Health 64: 12 1074–1079. 10.1136/jech.2009.099721 PMC2989158 19955098

[34] Kotloff K, Blackwelder W, Nasrin D, Nataro JP, Farag TH, et al (2012) The Global Enteric Multicenter Study (GEMS) of Diarrheal Disease in Infants and Young Children in Developing Countries: Epidemiologic and Clinical Methods of the Case/Control Study. Clinical Infectious Diseases 55: Supplement 4 S232–245. 10.1093/cid/cis753 PMC3502307 23169936

[35] Levine MM, Kotloff KL, Nataro JP, Muhsen K (2012) The Global Enteric Multicenter Study (GEMS): impetus, rationale, and genesis. Clinical Infectious Diseases 55: Supplement 4 S215–224. 10.1093/cid/cis761 PMC3502311 23169934

[36] Blum D, Feachem RG (1983) Measuring the impact of water supply and sanitation investments on diarrhoeal diseases: problems in methodology. International Journal of Epidemiology 12: 357–365. 6629626 10.1093/ije/12.3.357

[37] Schmidt WP, Arnold BF, Boisson S, Genser B, Luby SP, et al (2011) Epidemiological methods in diarrhoea studies – an update. International Journal of Epidemiology 40: 6 1678–1692. 10.1093/ije/dyr152 PMC3235024 22268237

[38] Biran A, Jenkins MW, Dabrase P, Bhagwat I (2011) Patterns and determinants of communal latrine usage in urban poverty pockets in Bhopal, India. Tropical Medicine and International Health 16: 7 854–862. 10.1111/j.1365-3156.2011.02764.x 21414114

[39] Tiimub BM, Forson MA, Obiri-Danso K, Rahaman IA (2009) Pointed gaps in the provision, quality, patronage and management of toilet facilities in Bawku East District; 34th WEDC International Conference; Addis Ababa, Ethiopia.

[40] Mukherjee N, Robiarto A, Saputra E, Wartono D (2012) Achieving and Sustaining Open Defecation Free Communities: Learning from East Java; WSP; Water and sanitation program.

[41] Oduro-Kwarteng S, Awuah E, Nyarko KB (2009) Shifting from public shared toilets to home toilets in urban settlements: implications of household demand in Kumasi, Ghana; 34th WEDC International Conference; Addis Ababa, Ethiopia.

