# Supplementary figures and images for: Shared Sanitation versus Individual Household Latrines: A Systematic Review of Health Outcomes
Source: PLoS One. 2014 Apr 17;9(4):e93300. doi: 10.1371/journal.pone.0093300 (PMC3990518; doi:10.1371/journal.pone.0093300)

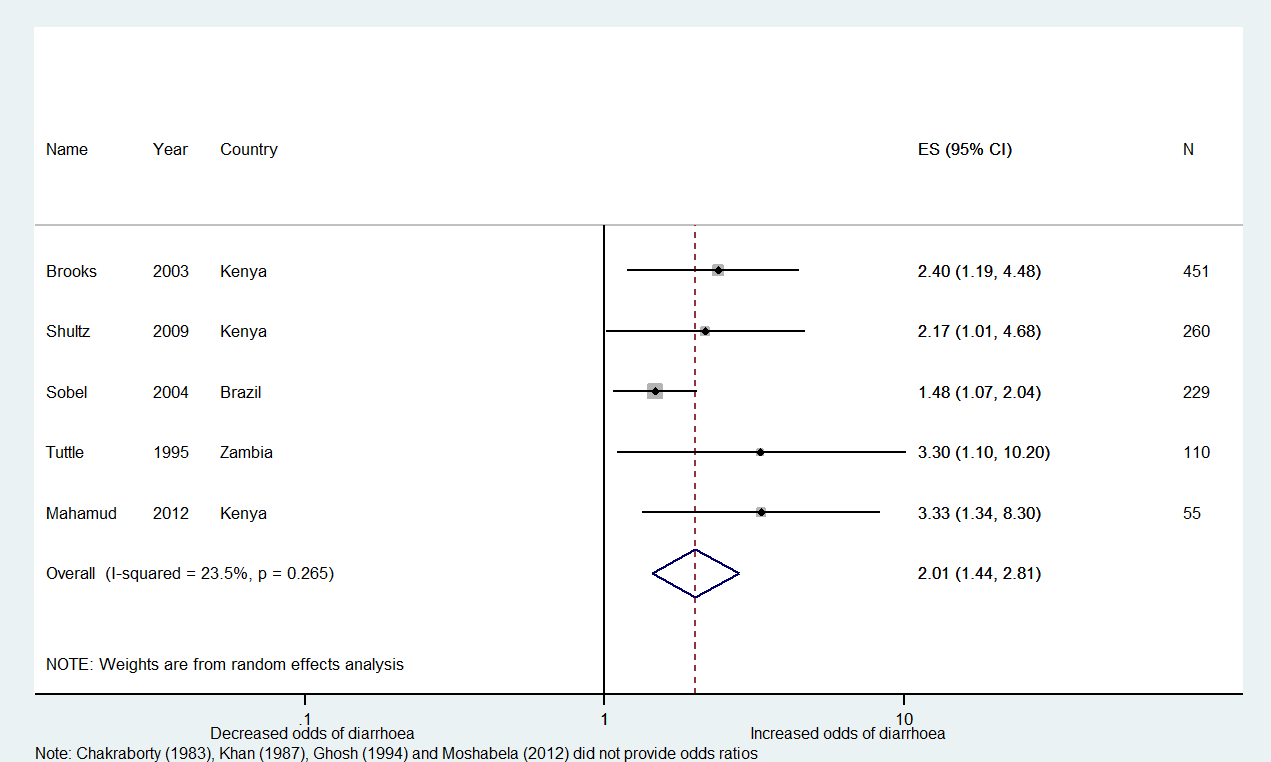

Supplement: Figure S1 — Sub group forest plot-Published data only. Image produced using Stata (Statacorp LP, TX USA). CI: Confidence Interval. ES: Effect size (Odds Ratio). (TIF) [file pone.0093300.s002.tif]

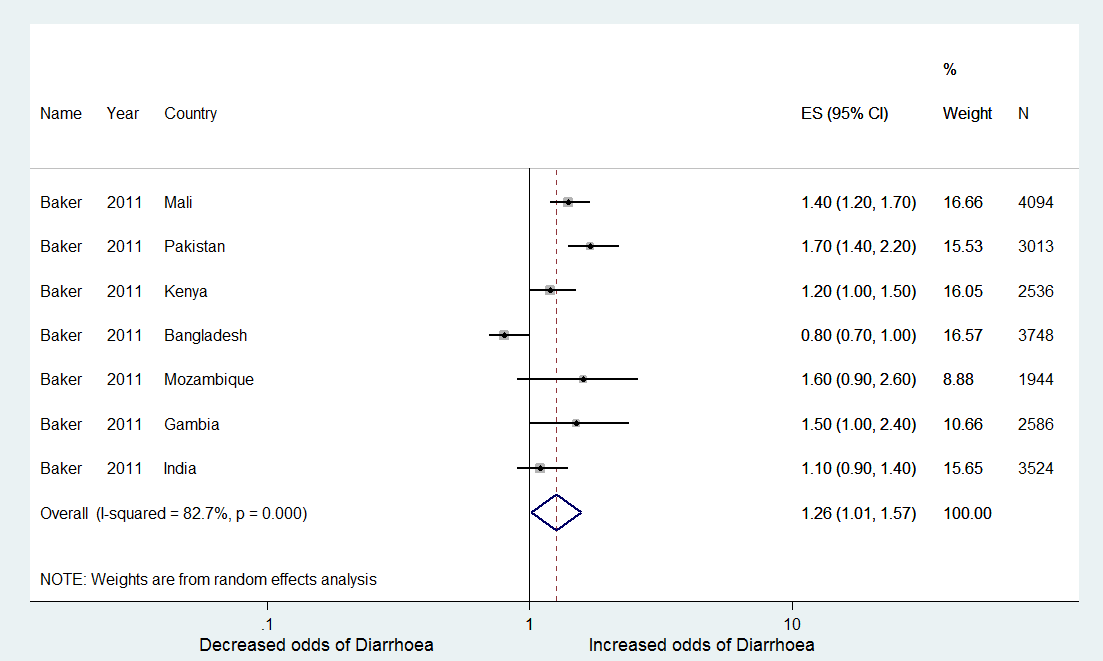

Supplement: Figure S2 — Sub group forest plot-Unpublished data only. Image produced using Stata (Statacorp LP, TX USA). CI: Confidence Interval. ES: Effect size (Odds Ratio). (TIF) [file pone.0093300.s003.tif]
