# Supplementary material for: Shared Sanitation versus Individual Household Latrines: A Systematic Review of Health Outcomes
Source: PLoS One. 2014 Apr 17;9(4):e93300. doi: 10.1371/journal.pone.0093300 (PMC3990518; doi:10.1371/journal.pone.0093300)
Supplement: Protocol S1 — (PDF) [file pone.0093300.s004.pdf]

Shared Sanitation - A Systematic Review

PROTOCOL

Marieke Heijnen, Oliver Cumming, Rachel Peletz, Joe Brown, Thomas Clasen

March 2012

This is a protocol for a systematic review on shared sanitation. The main objective of the review is to compare shared sanitation with individual household latrines. Outcomes of interest will include (i) health impact (diarrhoea, helminth infections, enteric fevers, trachoma, under-nutrition), (ii) intermediate outcomes that are related to exposure to disease pathogens (drinking water quality, flies, presence of faeces etc.), (iii) measure of sanitation uptake (latrine use, changes in open defecation, etc.), (iv) equity and other social impacts of sanitation. Analysis will focus on comparisons between individual household latrines use (or other practices) and shared sanitation, but we will also analyze studies that identify associations between only shared sanitation and the outcomes of interest.

BACKGROUND

Introduction

According to the United Nations Children's Fund (UNICEF) and the World Health Organization (WHO) an estimated 2.5 billion people lack access to improved sanitary facilities, such as a basic pit latrine, a toilet connected to a piped sewer system or septic tank, or a composting toilet [1]. In developing regions where people are most vulnerable to infection, only one in every two people has access to improved sanitation [2]. Only 41 per cent of people in sub-Saharan Africa and 30 per cent of people in Southern Asia have access to improved sanitary facilities- the remainder use unimproved facilities, share a facility or practice open defecation [1]. However, there are significant differences between the two regions: in sub-Saharan Africa 45 per cent of the population use either shared or unimproved facilities, and an estimated 25 per cent practice open defecation; whereas in Southern Asia, the proportion of the population using shared or unimproved facilities is much lower, and open defecation is the highest of any region (42 per cent)[1].

Though the global population in 2011 was about equally divided between urban and rural, the urban-rural disparities in sanitation are significant. Globally, 79 per cent of the urban population use an improved sanitation facility, compared to 47 per cent of the rural population [1]. Despite significant and encouraging declines in open defecation since 1990, 1.1 billion people – 15 per cent of the world's population – still resort to the practice, the majority of whom live in rural areas [1].

According to the recent update on drinking water and sanitation, sanitation coverage is improving in almost every developing region. Despite this, it is unlikely that Target 10 of Goal 7 of the Millennium Development Goals (MDGs) - aiming to halve the proportion of people with access to basic sanitation by 2015- will be met [1]. Unless the pace of change in the sanitation sector can be accelerated, the MDG target may not be reached until 2026 [1].

Definitions of sanitation

In its broadest sense, sanitation deals with the safe collection, storage, treatment, and disposal, reuse or recycling of human excreta (faeces and urine), as well as the drainage, disposal, recycling and re-use of waste water and storm water, and household, industrial and hazardous waste [3].

The MDG target, which is expressed in terms of 'basic sanitation' follows a more comprehensive approach and also includes concepts of affordability, cultural acceptability and environmental sustainability [4].

The United Nations Millennium Taskforce on Water and Sanitation attempted to consolidate these definitions, defining basic sanitation 'as the lowest-cost option for securing sustainable access to safe, hygienic, and convenient facilities and services for excreta and sullage disposal that provide privacy and dignity, while at the same time ensuring clean and healthful living environment both at home and in the neighbourhood of users [3].

The MDG definition is context specific- in dispersed, low-income rural areas it may include a simple pit latrine, whilst in congested urban slums with a reliable water service, household-based solutions would be deemed inadequate and low-cost sewerage systems would be necessary to ensure the proper collection, treatment, and disposal or reuse of excreta and household wastewater [3].

The Joint Monitoring Programme (JMP) for Water Supply and Sanitation defines "improved sanitation" and "unimproved sanitation" in terms of the facilities available for the disposal of human excreta. Improved sanitation facilities includes a private flush or pour-flush toilet or latrine connected to a piped sewer system or septic system, a simple pit latrine with a slab, a ventilated improved pit latrine (VIP) or a composing toilet. Unimproved sanitation includes any other flush or pour-flush latrine, an open pit latrine, bucket latrine, a hanging latrine, any public or shared facility or open defecation [2].

In locations or situations where there is insufficient space to construct a private sanitary facility, such as in densely populated urban areas, people often rely on public or shared facilities [2]. Shared sanitation facilities as defined for MDG monitoring purposes are facilities of an otherwise improved type that are either public or shared between two or more households [2]. This includes toilets shared between a group of households in a single building or plot, one shared in a community by several households as well as public toilets which are open to anybody and will often include some form of payment [5]. Households that use shared or public facilities are not included in the population defined as using an improved sanitation facility, and as such do not meet the JMP criterion for improved sanitation [6]. The reason stems from concerns that shared facilities are unacceptable both in terms of cleanliness (toilets may not be hygienic and fully separate human waste from contact with users) and accessibility (facilities may not be available at night, or used by children, for instance).

Among the different regions, using a shared facility is most common in sub-Saharan Africa and Eastern Asia (both 19 per cent) and particularly common in certain sub-Saharan African counties such as Ghana (58 per cent), Congo and Gabon (both 34 per cent)[1].

However, JMP recognizes that, globally, the number of people using shared sanitation is growing: The number of users has increased by 425 million since 1990 – increasing from 6 per cent of the global population to 11 per cent in 20 years. In many countries, particularly in crowded urban areas, shared sanitation is the only viable option for those wishing to avoid open defecation; in rural areas, families often keep costs down by sharing latrines between one or more households with family ties. A JMP task force on sanitation is exploring the issue of shared sanitation as part of its mandate [1].

Sanitation and health

Approximately 6.3 per cent of deaths and 9.1 per cent of DALYs (disability-adjusted life years) worldwide are attributable to unsafe water, sanitation and hygiene [7]. While the biological association between diarrhoea and exposure to human faeces is well established, there is little rigorous epidemiological evidence of the effectiveness of sanitation interventions to prevent disease [8]. Much of the evidence of the effectiveness and mechanisms of improved sanitation to prevent diarrhoea derives from observational studies [9-11]. A recent Cochrane review noted that there was some evidence of interventions to improve excreta disposal which were effective in preventing diarrhoeal diseases, however the quality of the evidence was deemed poor [8].

Inadequate water and sanitation are linked to a broad range of health problems; according to the 2011 Human Development Report, billions of people are affected by parasitic diseases: 1.5 billion with *ascaris*, 740 million with hookworm, 200 million with schistosomiasis and 40–70 million with liverfluke. These infections as well as hepatitis, typhoid and polio can be avoided through safe excreta disposal and other hygienic behaviours [12]. Half of all malnutrition is attributable to environmental factors, particularly poor water, and sanitation and hygiene [13]. Malnutrition from these causes is responsible for some 70,000 child deaths a year, while underweight children are more vulnerable to infectious disease and less likely to recover fully when they do fall sick [7].

It is estimated that 15 per cent of deaths in children younger than 5 years worldwide are caused by diarrhoea[14]. Diarrhoea is known to be caused by a wide variety of bacterial, viral, and protozoan pathogens excreted in the faeces of humans and animals. The infectious agents associated with diarrhoeal disease are transmitted chiefly through the faecal-oral route [15]. The importance of individual pathogens varies between settings, seasons and conditions. These pathogens may be transmitted through the ingestion of contaminated food, water, or other beverages, by person-to-person contact and by direct or indirect contact with infected faeces. Due to the different pathways, environmental interventions for the prevention of diarrhoeal disease typically include steps to improve the proper disposal of human faeces (sanitation) as well as improving water quality [16], water quantity and access, and promoting hand washing and other hygienic practices [17, 18] Many studies have reported results of interventions to reduce illness through improvements in drinking water, sanitation facilities and hygiene practices- though limited data is available, it has been suggested that sanitation interventions can significantly reduce diarrhoeal illness, with a pooled relative risk of 0.68 [19]. Excreta disposal is associated with a 36 per cent reduction in diarrhoea morbidity [20], a figure which is confirmed in the more recent review of data [21]. It was noted however, that the data remains very limited and the few available studies are not of high quality.

Shared sanitation and health

There is evidence that shared sanitation is associated with poorer health outcomes compared to individual household latrines, including lower birth weight [22] and higher perinatal mortality [23], helminth infection [24] and risk of polio during an outbreak [25]. The exclusion of shared sanitation in the JMP definition is based primarily on evidence suggesting lower levels of use of these facilities versus individual household latrines, possibly due to poor maintenance of the shared facilities. When Montgomery and colleagues [26, 27] looked more closely at their data from a sanitation intervention (latrines) to prevent trachoma, they found no difference in rates of infection among those with shared sanitation provided they controlled for use. This is consistent with the hypothesis that Clasen and colleagues are pursuing with support from the Bill & Melinda Gates Foundation, 3ie and the SHARE Consortium in Orissa, India, that securing widespread use (and not only coverage) of latrines is the key driver in achieving health gains from sanitation [28].

Non-health outcomes and sanitation

Without access to sanitary facilities people are forced to defecate in fields, plastic bags, ditches and buckets. Besides the considerable public health risk associated with this, it is accompanied by loss of dignity and considered a source of insecurity, especially for women. In settings where people live in very close proximity to one another, such as in urban slums, having no safe, private sanitation facilities means going the whole day without relieving oneself and then risking exposure at night- a humiliating, stressful and uncomfortable daily routine that can damage health [3]. Recent research [29] in various urban slums in Delhi, India highlighted that women were fearful of sexual violence when using public toilets, when defecating in the open and in public spaces in general. In one area, community toilet blocks were not mentioned as dangerous in themselves but the routes to the toilet blocks were associated with sexual violence. It was reported that women and girls faced lewd remarks, physical gestures and rape when they relieved themselves in the bushes- as a result some women attempted to build toilets in their homes [29].

Though difficult to quantify, the pride, social status and comfort which comes with access to a clean and safe latrine has been reported by many new latrine users [3, 30]. In addition to enhancing dignity, privacy and safety- especially for women and girls-, improved sanitation benefits the economy- every dollar spent on sanitation generates economic benefits worth around nine more; sanitary disposal of human excreta also offers certain benefits for the environment [31].

AIMS AND OBJECTIVES

The JMP Taskforce for Water Supply and Sanitation met in 2010 to discuss the decision to consider shared and public sanitation facilities as “not improved”[6]. It was noted during this meeting that a strong evidence base is lacking. As a result, the JMP together with the LSHTM-based, DFID-funded SHARE Research Consortium (http://www.shareresearch.org) commissioned this review as part of an overall research plan aimed at strengthening of this evidence base. This protocol describes the methodology for a systematic literature review on the impact of shared sanitation. Both health and non-health outcomes will be explored. Analysis will focus on comparisons with individual household latrines, but studies related shared sanitation alone will also be considered.

The main objective of the review is to compare shared sanitation with individual household latrines. Outcomes of interest include (i) health impact (diarrhoea, helminth infections, enteric fevers, trachoma etc.), (ii) intermediate outcomes that are related to exposure to disease pathogens (drinking water quality, flies, presence of faeces), (iii) measure of sanitation uptake (latrine use, changes in open defecation, etc.), and iv) equity and other social impacts of sanitation. Analysis will focus on comparisons with individual household latrines, but we will also analyze studies that identify associations between shared sanitation and the outcomes of interest.

CRITERIA FOR CONSIDERING STUDIES FOR THIS REVIEW

Types of studies

Observational designs as well as intervention studies will be included in the review.

Types of participants

Infants, children and adults in low- and middle-income settings.

Types of exposure

All domestic excreta-disposal facilities that are shared by more than one household. This includes any type of sanitation facilities, whether on-site (e.g., pit latrines, toilets connected to septic systems) or reticulated (e.g., toilets connected to sewerage system) regardless of whether they meet the JMP definition of "improved" or "unimproved", though we will do sub-group analysis on such characterization. The sanitation facilities may be owned or maintained individually by one or more households or by a commercial or government entity. However, sanitation facilities designed primarily for use by householders when they are away from the home, such as schools, markets, train or bus stations, city streets or other public places, are excluded. We will include sanitation facilities that combine improvements in excreta disposal with other environmental interventions such as improvements in water quantity or access, water quality or hygiene practices, but will again conduct sub-group analysis on these facilities.

Types of outcome measures

Health outcomes

- Diarrhoeal diseases
- Enteric infection, regardless of microbial agent
- Nutritional status, mainly measured through anthropometry
- Helminthiasis
- Trachoma
- Dracunculiasis
- Enteric fevers such as typhoid
- Stress, psychological

Non-health outcomes

- Knowledge, attitudes and practices of exposed population
- Utilisation, adherence, compliance, uptake of facilities
- Condition, operation and maintenance of facilities
- Utilisation by gender
- (Sexual) violence
- Cost
- Social impact
- Equity

Intermediate outcomes related to exposure to disease pathogens

- Water access
- Water quantity
- Water quality
- Hand contamination
- Flies
- Hand washing behaviour
- Hygiene behaviour

SEARCH STRATEGY FOR IDENTIFICATION OF STUDIES

We will attempt to identify all relevant studies regardless of language or publication status (published, unpublished, in press and in progress), using the following search strategy (individual search terms can be found in table 1):

```
1. SANITATION/ or SEWAGE/ or WASTE DISPOSAL, FLUID/ or REFUSE DISPOSAL/ or
   TOILET FACILITIES/ or (SANITA* or (EXCRETA adj2 DISPOSAL) or TOILET* or
   LATRINE* or SEWERAGE or (SEWAGE adj2 DISPOSAL) or (WASTE adj2 DISPOSAL) or
   (FE*CES adj2 DISPOSAL)).ti,ab.

2. (SHARED or COMMU* or COMMON or PUBLIC or IMPROVE* or SLUM* or COLLECTIVE or
   SAFE).ti,ab.

3. DIARRHEA, INFANTILE/ or DIARRHEA/ or (DIARRH*EA or DIARRH*EAL
   DISEASE*).ti,ab. or CHOLERA/ or GASTROINTESTINAL DISEASES/ or INTESTINAL
   DISEASES,PARASITIC/ or TYPHOID FEVER/ or PARATYPHOID FEVER/ or NEGLECTED
   DISEASES/ or STRESS,PSYCHOLOGICAL/ or SEX OFFENSES/ or VIOLENCE/ or INFANT
   NUTRITION DISORDER/ or CHILD NUTRITION DISORDER/ or CHILD WELFARE/ or
   INFANT WELFARE/ or INFANT NUTRITION DISORDER/ or CHILD NUTRITION DISORDER/
   or GENDER IDENTITY/ or COST ANALYSIS/ or SOCIAL CHANGE/ or HYGIENE/ or
   HEALTH PROMOTION/ or HANDWASHING/ or WATER QUALITY/ or (COST or UTILI*ATION
   or (OPERATION adj2 MAINTENANCE) or ADHERENCE or COMPLIANCE or MAINTENANCE
   or UPTAKE or EQUITY or (WATER adj2 QUANTITY) or (WATER adj2 ACCESS)).ti,ab.

4. 1 and 2

5. 3 and 4
```

Databases

The key terms for the search can be found in Table 1.

The following databases will be searched using OvidSP (Ovid Technologies 2012):

MEDLINE, EMBASE, CAB Abstracts, Global Health, HMIC, Social Policy & Practice

The following databases will be searched using Virtual Health Library:

DESASTRES, LEYES, LILACS, MedCarib, REPIDISCA

The remaining databases will be searched separately:

BASE, CEHA Database, Chicano Database, CINAHL Plus, ERIC, HISA, IBSS, Library Information Science & Technology Abstracts, TRIP Database, WPRIM, Web of Science, Africa-Wide Information

Grey literature, theses and survey datasets

Additionally, grey literature, theses and survey datasets will be searched using the following sources:

Dissertations & Theses, EThoS, Index to Theses of the British Isles, ELDIS, NBER Working Papers, New York Academy of Medicine Grey Literature Report, Open Grey, ReliefWeb, ESDS International, Cochrane Infectious Diseases group's trial register, Cochrane central register of controlled trials (CENTRAL) published in The Cochrane Library

Conference proceedings

Various conference proceedings will be searched for relevant abstracts, including, but not limited to WEDC (Loughborough University), IRC International Water and Sanitation Centre, World Bank, and German Agency for International Cooperation (GIZ).

Researchers, organisations and companies

Individual researchers working in the field will be contacted- these include the Water Sanitation and Health programme of the World Health Organisation; the World Bank Water and Sanitation programme; UNICEF Water Sanitation and Hygiene (WASH) programme; Environmental Health project (EHP) at USAID; IRC International Water and Sanitation centre; Foodborne and Diarrhoeal Disease Branch, Division of Bacterial and Mycotics Diseases, Centres for Disease Control and Prevention (CDC); UK Department for International Development (DFID); a variety of Non Governmental Organisation working in the field of sanitation including Plan International, WorldVision, WaterAid and Oxfam.

Reference lists

We will also check the reference lists of all studies identified by the above methods

METHODS OF THE REVIEW

Selection of studies

Marieke Heijnen (MH) and Oliver Cumming (OC) will independently review the titles and abstracts resulting from the search and select all studies that potentially fall within the inclusion criteria for the review. After obtaining full copies of all such studies, we will independently determine if the study meets such inclusion criteria. Where there is agreement, the studies will either be included or excluded. Where there is no agreement, Thomas Clasen (TC) will be consulted to make the final decision on eligibility for inclusion. Any studies that MH or OC proposed to include but which were ultimately determined by TC not to be included will be identified together with the reason for exclusion in the 'Characteristics of excluded studies'.

During the search, a list of excluded documents will be maintained with reasons for exclusion. In addition, careful documentation will be maintained on the data source, search strategy and date of search for the included documents.

Data extraction

Data from all relevant articles will be extracted by MH. The data extraction forms will be based on the data collection form from the Cochrane Effective Practice and Organisation of Care (EPOC) Group and Cochrane Public Health Group, modified for use in this review [32, 33]. Quality criteria questions for the different study designs will be built into this form. Data will be extracted, and included in the 'characteristics of included studies', on the following:

- Study design and sample size
- Method of participant selection
- Study duration
- Details of participants
- Study setting (country and urban/rural)
- Description of intervention or exposure (type of sanitary facility used and whether it is shared between households, communities, or a public facility; any promotional campaigns the population may have been exposed to)
- Water, sanitation, and hygiene characteristics (water source, water quality, sanitation facilities, hygiene practices)
- Definition and practices of control group

- Unit of randomization (and whether study adjusted for clustering if randomization is not individual)
- Unit of analysis
- Description of outcomes (including case definition of health outcomes, use and maintenance of the facilities, social impact and knowledge, attitudes and practices of the exposed population, microbiological data, observational data on hygienic conditions compared between the two options, or use frequency data- see table 1 for full list)
- Type of data available (microbiological data, observational data on hygienic conditions, frequency-of-use data)
- Intervention coverage (before and after implementation)
- Intervention uptake
- Information on intervention cost
- Publication status
- Quality control (see assessment of risk of bias below)

Multiple papers reporting results from one study will be treated as one study. We will develop data extraction forms based on the Cochrane Effective Practice

Assessment of methodological quality

MH and OC will independently assess the methodological quality of the studies. The risk of bias of the included studies will be assessed using the EPOC risk of bias tool for studies with a separate control group. This includes the standard Cochrane risk of bias tool items to assess file domains of bias: selection performance, attrition, detection and reporting, as well as additional items to assess the risk of selection bias.

The EPOC tool specifies the following criteria for studies with a separate control group:

- *Was the allocation sequence adequately generated?* (random=adequate, non-random inadequate)
- *Was the allocation adequately concealed?* (centralized randomization scheme, on-site computer system, sealed envelopes= adequate, controlled before/after studies=no)
- *Were baseline outcome measurements similar?* (balances or appropriately adjusted=yes, imbalanced and inadequately adjusted for=no)
- *Were baseline characteristics similar?* We will consider diarrheal morbidity, age, socioeconomic status, water quality, water sources, hygiene practices, and sanitation facilities
- *Were incomplete outcome data adequately addressed?* For example, examining loss-to-follow-up and missing data (adequate if LTFU if ≤15%)
- *Was knowledge of the allocated interventions adequately prevented during the study?* (blinding=yes, non-blinded=no)
- *Was the study adequately protected against contamination?* (unlikely that control group received intervention=yes, control group likely received intervention=no)
- *Was the study free from selective outcome reporting? (*all outcomes in methods are reported=yes, important outcomes omitted from results=no)
- *Was the study free from other risks of bias?* Specifically, we will examine whether the control and intervention groups were assessed at similar points in time.

There is a similar EPOC tool for interrupted time series studies if the same population is examined before/after an intervention.

Dependent on the type of observational studies which may be found, the STROBE statement criteria will be considered [34].

For purely qualitative studies, there is no single validated checklist to use for all types of qualitative studies [32]. The following criteria have been suggested for assessing quality common to all qualitative research:

- Method appropriate to research question
- An explicit l ink to theory
- Clearly stated aims and objectives
- A clear description of context
- A clear description of sample
- A clear description of fieldwork methods
- Some validation of data analysis
- Inclusion of sufficient data to support interpretation[32]

Assessment of reporting biases

If there are adequate number of studies (≥10), bias will be assessed using funnel plots plotting the effect size against the standard error of effect with a measure of heterogeneity (I^2 statistic). If study sizes are too small to calculate standard errors, we will try to plot the effect size against the cluster size.

Data analysis

The data from the included studies will be summarised and tabulated by MH and TC. We will report statistically significant and non-significant outcomes according to type of study design. In the case of insufficient data, a narrative synthesis will be conducted and in this situation we anticipate that studies will be grouped by either outcome type or intervention type. We will attempt to include a summary of findings table to provide information about the primary outcomes, effect sizes, process and implementation factors, cost of intervention and quality of the information.

Subgroup analysis and investigation of heterogeneity

Where sufficient data are available we will perform additional sub-group analyses to compare outcomes by the following characteristics

- Sanitation type (improved vs. unimproved, on-site vs. sewerage)
- Community location (urban, rural)
- Number of households using the facilities
- Level of latrine coverage
- Level of latrine maintenance
- Consistency of use
- Water quality
- Water quantity
- Water access
- Hygiene practices

We expect to find substantial diversity in the methodological approaches, as well as the exposures and outcomes. This includes (i) heterogeneity of exposure- people use different types of sanitary facilities, both in terms of design and the number of people using the facilities, (ii) heterogeneity of outcome- the different health outcomes specified have different etiologies and transmission routes; the non-health outcomes will vary considerably in terms of definitions used and units of measurement, and (iii) heterogeneity of setting – this relates both to the physical setting (rural, urban, peri-urban, formal, and informal) as well as the climate and season. In addition, the actual transmission of diseases depends on the infection intensity (populations with high worm-load but low transmission versus populations with low worm-load but high transmission). Lastly, the setting of the actual interventions of interest will be heterogeneous- water quality, availability and access, level of personal hygiene, knowledge and attitudes in the community.

All of these issues will be considered and the evidence base will determine the prospects for the type of (statistical or non-statistical) aggregation.

Table 1- Key search terms

**Interventions/Exposure**	**Outcomes**
Sanita*	Diarrhea, Infantile/
Excreta Disposal	Diarrhea/
Fe*ces disposal	Diarrh*ea
Toilet*	Diarrh*eal disease
Latrine*	Cholera/
TOILET FACILITIES/	Infant welfare/
SANITATION/	Child welfare/
WASTE DISPOSAL,FLUID/	Hygiene/
Waste disposal	Health promotion/
SEWAGE/	Handwashing/
Sewerage	Infant nutrition disorder/
Sewage disposal	Child nutrition disorder/
REFUSE DISPOSAL/	Water quantity
Shared	Water access
Commu*	Equity
Common	Uptake
Public	Adherence
Improve*	Compliance
Slum*	Maintenance
Collective	Cost
Safe	Cost analysis/
	Operation and maintenance
	Utili*ation
	Stress, Psychological/
	Gender identity/
	VIOLENCE/
	SEX OFFENSES/
	Social change/
*indicates truncation<br>/indicates MESH term	

REFERENCES

1. WHO/UNICEF, *Progress on Drinking Water and Sanitation: 2012 Update*, in *WHO/UNICEF Joint Monitoring Programme for Water Supply and Sanitation*2012.
2. WHO/UNICEF, *Progress on Sanitation and Drinking-Water: 2010 Update*, W.H.O.a. UNICEF, Editor 2010.
3. United Nations Millennium Project, *Health, Dignity, and Development: What Will it Take?*, U.N.M. Project, Editor 2005, Task Force on Water and Sanitation.
4. United Nations, *Report on the World Summit on Sustainable Development*, 2002: Johannesburg.
5. Water and Sanitation for the Urban Poor, *When are communal or public toilets an appropriate option?* , in *Topic Brief*, W.S.f.t.U. Poor, Editor 2011.
6. JMP Taskforce *Joint Monitoring Programme Technical Task Force-Meeting on Sanitation and Methods for Estimating Progress*, 2010, UNICEF/WHO.
7. Prüss-Üstün, A., et al., *Safer water, Better health- Costs, benefits and sustainability of interventions to protect and promote health*, W.H. Organization, Editor 2008: Geneva.
8. Clasen TF, B.K., Schmidt WP, Boisson S, Fung ICH, Jenkins MW, Scott B, Sugden S, Cairncross S, *Interventions to improve disposal of human excreta for preventing diarrhoea (Review).* The Cochrane Library, 2010(6).
9. Barreto, M.L., et al., *Effect of city-wide sanitation programme on reduction in rate of childhood diarrhoea in northeast Brazil: assessment by two cohort studies.* The Lancet, 2007. **370**(9599): p. 1622-1628.
10. Green ST, S.M., Casman EA, *Determinants of national diarrheal disease burden.* Environ Sci Technol., 2009. **43**(4): p. 993-9.
11. Genser, B., Strina A, dos Santos LA, Teles CA, Prado MS, Cairncross S, *Impact of a city-wide sanitation intervention in a large urban centre on social, environmental and behavioural determinants of childhood diarrhoea: analysis of two cohort studies.* Int J Epidemiol., 2008. **37**(4): p. 831-40.
12. UNDP, *Sustainability and Equity: A Better Future for All*, in *Human Development Report*2011, United Nations Development Programme: New York.
13. Prüss-Üstün A, C.C., *Preventing disease through healthy environments. Towards an estimate of the environmental burden of disease*, W.H. Organisation, Editor 2006.
14. Black, R.E., et al., *Global, regional, and national causes of child mortality in 2008: a systematic analysis.* The Lancet, 2010. **375**(9730): p. 1969-1987.
15. Byers KE, G.R., Farr BM, *Fecal-oral tranmission*, in *Epidemiologic Methods for the Study of Infectious Diseases*, W.D. Thomas JC, Editor 2001, Oxford University Press: Oxford. p. 228-48.
16. Clasen T, R.I., Rabie T, Schmidt W, Cairncross S, *Interventions to improve water quality for preventing diarrhoea.* Cochrane Database Syst Rev., 2006. **3**: p. CD004794.
17. Curtis V, C.S., *Effect of washing hands with soap on diarrhoea risk in the community: a systematic review.* Lancet Infect Dis., 2003. **3**(5): p. 275-81.
18. Ejemot RI, E.J., Meremikwu MM, Critchley JA, *Hand washing for preventing diarrhoea.* Cochrane Database Syst Rev., 2008(1): p. CD004265.
19. Fewtrell, L., et al., *Water, sanitation, and hygiene interventions to reduce diarrhoea in less developed countries: a systematic review and meta-analysis.* The Lancet Infectious Diseases, 2005. **5**(1): p. 42-52.
20. Esrey SA, P.J., Roberts L, Shiff C., *Effects of improved water supply and sanitation on ascariasis, diarrhoea, dracunculiasis, hookworm infection, schistosomiasis, and trachoma.* Bull World Health Organ, 1991. **69**(5): p. 609-21.
21. Cairncross, S., et al., *Water, sanitation and hygiene for the prevention of diarrhoea.* Int J Epidemiol., 2010. **39**(Suppl 1): p. i193-205.
22. Olusanya, B.O. and G.E. Ofovwe, *Predictors of preterm births and low birthweight in an inner-city hospital in sub-Saharan Africa.* Matern Child Health J, 2010. **14**(6): p. 978-86.
23. Golding, J., et al., *Associations between social and environmental factors and perinatal mortality in Jamaica.* Paediatr Perinat Epidemiol, 1994. **8 Suppl 1**: p. 17-39.

24. Curtale, F., et al., *Different patterns of intestinal helminth infection among young workers in urban and rural areas of Alexandria Governorate, Egypt.* Parassitologia, 1998. **40**(3): p. 251-4.
25. Kim-Farley, R.J., et al., *Outbreak of paralytic poliomyelitis, Taiwan.* Lancet, 1984. **2**(8415): p. 1322-4.
26. Montgomery, M.A., M.M. Desai, and M. Elimelech, *Comparing the effectiveness of shared versus private latrines in preventing trachoma in rural Tanzania.* Am J Trop Med Hyg, 2010. **82**(4): p. 693-5.
27. Montgomery, M.A., M.M. Desai, and M. Elimelech, *Assessment of latrine use and quality and association with risk of trachoma in rural Tanzania.* Trans R Soc Trop Med Hyg, 2010. **104**(4): p. 283-9.
28. Clasen, T., *Developing the Evidence to Assess the Health Impact and Use of Shared Sanitation versus Individual Household Latrines*, in *Research Proposal*2011.
29. Lennon, S. *Fear and Anger: Perceptions of risks related to sexual violence against women linked to water and sanitation in Delhi, India* SHARE Briefing note, 2011.
30. UNDP, *Beyond scarcity:Power, poverty and the global water crisis*, in *Human Development Report 2006*, M.d.C.a.C.T. Bruce Ross-Larson, Editor 2006, United Nations Development Programme: New York.
31. *Keeping sanitation in the international spotlight.* The Lancet, 2008. **371**(9618): p. 1045.
32. Armstrong R, W.E., Jackson N, Ol iver S, Popay J, Shepherd J, Petticrew M, Anderson L, Bai l ie R, Brunton G, Hawe P, Kristjansson E, Naccarel la L, Norris S, Pienaar E, Roberts H, Rogers W, Sowden A, Thomas H. *Guidelines for Systematic reviews of health promotion and public health interventions*. 2007 [cited 2012 16 March]; Version 2:[Available from: http://ph.cochrane.org/sites/ph.cochrane.org/files/uploads/Guidelines%20HP_PH%20reviews.pdf.
33. Collaboration, C. *Risk of bias*. EPOC Author Resources 2009; Available from: http://epoc.cochrane.org/epoc-author-resources.
34. STROBE Statement- Strengthening the Reporting of Observational studies in Epidemiology. 2009 [cited 2012 March 16]; Available from: http://www.strobe-statement.org/index.php?id=strobe-home.
