# Supplementary material for: Shared Sanitation versus Individual Household Latrines: A Systematic Review of Health Outcomes
Source: PLoS One. 2014 Apr 17;9(4):e93300. doi: 10.1371/journal.pone.0093300 (PMC3990518; doi:10.1371/journal.pone.0093300)
Supplement: Table S1 — Key search terms. (DOCX) [file pone.0093300.s005.docx]

**Table S1. Key search terms**

| **Search strategy** | |
| --- | --- |
| (**BLOCK 1 *AND* BLOCK 2) *AND* (BLOCK 3 *OR* BLOCK 4)** | |
| **BLOCK 1** | **BLOCK 2** |
| Sanita* | Shared |
| Excreta Disposal | Commu* |
| Fe*ces disposal | Common |
| Toilet* | Public |
| Latrine* | Improve* |
| TOILET FACILITIES/ | Slum* |
| SANITATION/ | Collective |
| WASTE DISPOSAL,FLUID/ | Safe |
| Waste disposal |  |
| SEWAGE/ |  |
| Sewerage |  |
| Sewage disposal |  |
| REFUSE DISPOSAL/ |  |
| **BLOCK 3** | **BLOCK 4** |
| Diarrh*eal disease | Water access |
| Cholera/ | Equity |
| Infant welfare/ | Uptake |
| Child welfare/ | Adherence |
| Hygiene/ | Compliance |
| Health promotion/ | Maintenance |
| Hand washing/ | Cost |
| Infant nutrition disorder/ | Cost analysis/ |
| Child nutrition disorder/ | Operation and maintenance |
| Water quantity | Utili*ation |
| Diarrhea, Infantile/ | Stress, Psychological/ |
| Diarrhea/ | Gender identity/ |
| Diarrh*ea | Violence/ |
|  | Sex Offenses/ |
|  | Social change/ |
| *indicates truncation /indicates MESH term | |
