# Supplementary material for: Shared Sanitation versus Individual Household Latrines: A Systematic Review of Health Outcomes
Source: PLoS One. 2014 Apr 17;9(4):e93300. doi: 10.1371/journal.pone.0093300 (PMC3990518; doi:10.1371/journal.pone.0093300)
Supplement: Table S2 — Search strategy as performed in OVID databases. (DOCX) [file pone.0093300.s006.docx]

**Table S2. Search strategy as performed in OVID databases**

| 1. SANITATION/ or SEWAGE/ or WASTE DISPOSAL, FLUID/ or REFUSE DISPOSAL/ or TOILET FACILITIES/ or (SANITA* or (EXCRETA adj2 DISPOSAL) or TOILET* or LATRINE* or SEWERAGE or (SEWAGE adj2 DISPOSAL) or (WASTE adj2 DISPOSAL) or (FE*CES adj2 DISPOSAL)).ti,ab. 2. (SHARED or COMMU* or COMMON or PUBLIC or IMPROVE* or SLUM* or COLLECTIVE or SAFE).ti,ab. 3. DIARRHEA, INFANTILE/ or DIARRHEA/ or (DIARRH*EA or DIARRH*EAL DISEASE*).ti,ab. or CHOLERA/ or GASTROINTESTINAL DISEASES/ or INTESTINAL DISEASES,PARASITIC/ or TYPHOID FEVER/ or PARATYPHOID FEVER/ or NEGLECTED DISEASES/ or STRESS,PSYCHOLOGICAL/ or SEX OFFENSES/ or VIOLENCE/ or INFANT NUTRITION DISORDER/ or CHILD NUTRITION DISORDER/ or CHILD WELFARE/ or INFANT WELFARE/ or INFANT NUTRITION DISORDER/ or CHILD NUTRITION DISORDER/ or GENDER IDENTITY/ or COST ANALYSIS/ or SOCIAL CHANGE/ or HYGIENE/ or HEALTH PROMOTION/ or HANDWASHING/ or WATER QUALITY/ or (COST or UTILI*ATION or (OPERATION adj2 MAINTENANCE) or ADHERENCE or COMPLIANCE or MAINTENANCE or UPTAKE or EQUITY or (WATER adj2 QUANTITY) or (WATER adj2 ACCESS)).ti,ab. 4. 1 and 2 5. 3 and 4 6. Limit 5 to human |
| --- |
|  |
