# Supplementary material for: Shared Sanitation versus Individual Household Latrines: A Systematic Review of Health Outcomes
Source: PLoS One. 2014 Apr 17;9(4):e93300. doi: 10.1371/journal.pone.0093300 (PMC3990518; doi:10.1371/journal.pone.0093300)
Supplement: Table S3 — Methodological quality. (DOCX) [file pone.0093300.s007.docx]

**Table S3. Methodological quality**

| **STROBE STATEMENT CRITERIA** | | **Authors** | **Brooks** | **Chakraborty** | **Chandiwana** | **Curtale** | **Ghosh** | **Golding** | **Hall** | **Khan** | **Kim-Farley** | **Mahfouz** | **Montgomery** | **Munoz** | **Olusanya** | **Phiri** | **Shultz** | **Sobel** | **Tsikuka** | **Tuttle** | **Moshabela** | **Karkey** | **Mahamud** |
| --- | --- | --- | --- | --- | --- | --- | --- | --- | --- | --- | --- | --- | --- | --- | --- | --- | --- | --- | --- | --- | --- | --- | --- |
|  |  | **Description** |  |  |  |  |  |  |  |  |  |  |  |  |  |  |  |  |  |  |  |  |  |
| 1 | Title and Abstract | **a)** Indicate the study's design with a commonly used term in the title or abstract  **b)** provide in the abstract, an informative and balanced summary of what was done and what was found | + | + | + | + |  | + | + | + | + | + | + | + | + | + | + | + | + | + | + | + | + |
| 2 | Background/  rationale | Explain the scientific background and rationale for the investigation being reported | **+** | **+** | **+** | **+** | **+** | **+** | **+** | **+** | **+** | **+** | **+** | **+** | **+** | **+** | **+** | **+** | **+** | **+** | **+** | **+** | **+** |
| 3 | Objectives | State specific objectives, including any pre-specified hypotheses |  |  |  | **+** |  |  |  |  |  | **+** | **+** | **+** | **+** | **+** |  |  | **+** |  |  |  | **+** |
| 4 | Study Design | Present key elements of study design early in the paper |  |  | **+** | **+** | **+** | **+** | **+** | **+** |  |  | **+** | **+** | **+** | **+** | **+** | **+** |  | **+** | **+** | **+** | **+** |
| 5 | Setting | Describe the setting, location and relevant dates, including periods of recruitment, exposure, follow up and data collection | **+** | **+** | **+** | **+** | **+** | **+** | **+** | **+** | **+** | **+** | **+** | **+** | **+** | **+** | **+** | **+** | **+** | **+** | **+** | **+** | **+** |
| 6 | Participants | **a)** *cohort studies*- give the eligibility criteria and the sources and methods of selection of participants. Describe methods of follow up. *Case control studies-* give the eligibility criteria, and the sources and methods of case ascertainment and control selection. Give the rationale for the choice of cases and controls. *Cross sectional studies*- Give the eligibility criteria, and the sources and methods of selection of participants. **b)** *Cohort study*- For matched studies, give matching criteria and number of exposed and unexposed. *Case-control studies*- for matched studies, give matching criteria and the number of controls per case | **+** |  |  | **+** | **+** | **+** |  |  | **+** | **+** | **+** | **+** | **+** | **+** | **+** | **+** | **+** | **+** | **+** | **+** | **+** |
| 7 | Variables | Clearly define all outcomes, exposures, predictors, potential confounders and effect modifiers. Give diagnostic criteria, if applicable | **+** | **+** | **+** | **+** |  | **+** | **+** | **+** | **+** | **+** |  | **+** | **+** | **+** | **+** | **+** | **+** | **+** | **+** | **+** | **+** |
| 8 | Data sources/ measurement | For each variable of interest, give sources of data and details of methods of assessment (measurement). Describe comparability of assessment methods if there is more than one group | **+** | **+** | **+** | **+** |  | **+** | **+** | **+** | **+** | **+** |  |  | **+** | **+** | **+** | **+** | **+** | **+** | **+** | **+** | **+** |
| 9 | Bias | Describe any efforts to address potential sources of bias |  |  |  |  |  |  |  |  |  |  | **+** |  | **+** |  | **+** | **+** |  |  |  |  |  |
| 10 | Study Size | Explain how the study size was derived |  |  |  |  |  |  |  |  | **+** |  |  | **+** |  | **+** |  |  | **+** |  |  |  |  |
| 11 | Quantitative variables | Explain how quantitative variables were handled in the analyses. If applicable, describe which groupings were chosen, and why | **+** |  |  |  |  | **+** |  |  |  |  |  |  | **+** | **+** | **+** | **+** | **+** | **+** | **+** | **+** | **+** |
| 12 | Statistical Methods | **a)** Describe all statistical methods, including those used to control for confounding **b)** Describe any methods used to examine subgroups and interactions **c)** Explain how missing data were addressed **d)** Cohort study- if applicable, explain how loss to follow up was addressed. Case control study, if applicable, explain how matching cases and controls was addressed. Cross-sectional - if applicable, describe analytical methods taking account of sampling strategy **e)** describe any sensitivity analysis | **+** |  | **+** | **+** |  | **+** | **+** |  | **+** | **+** | **+** | **+** | **+** | **+** | **+** | **+** | **+** | **+** | **+** | **+** | **+** |
| 13 | Participants | **a)** Report the nr of individuals at each stage of the study- e.g., numbers of potentially eligible, examined for eligibility, confirmed eligible, included in the study, completed follow-up and analyzed **b)** give reasons for nonparticipation at each state **c)** consider use of a flow diagram | **+** |  |  |  |  |  |  |  |  |  |  |  |  |  |  |  |  |  | **+** |  |  |
| 14 | Descriptive data | **a)** Give characteristics of study participants (e.g. demographic, clinical, social) and information on exposures and potential confounders **b)** Indicate the nr of participants with missing data for each variable of interest **c)** Cohort studies- summarize follow-up time (ie. average and total amount) | **+** |  | **+** |  | **+** | **+** | **+** | **+** | **+** |  |  | **+** | **+** | **+** | **+** | **+** | **+** | **+** | **+** | **+** |  |
| 15 | Outcome data | *Cohort studies*- report nrs of outcome events or summary measures over time, *case-control study-* report nrs in each exposure category, or summary measures of exposure, *cross-sectional study*- report nrs of outcome events or summary measures | **+** | **+** | **+** | **+** | **+** | **+** | **+** | **+** | **+** | **+** | **+** | **+** | **+** | **+** | **+** | **+** | **+** | **+** | **+** | **+** | **+** |
| 16 | Main results | **a)** give unadjusted estimates and, if applicable, confounder-adjusted estimates and their precision (ie. 95% ci.) Make clear which confounders were adjusted for, and why they were included **b)** Report category boundaries when continuous variables were categorised **c)** if relevant, consider translating estimates of relative risk into absolute risk for meaningful time period | **+** |  |  |  |  | **+** |  |  |  | **+** | **+** | **+** | **+** | **+** | **+** | **+** | **+** | **+** | **+** | **+** | **+** |
| 17 | Other analyses | Report other analyses done, ie. Analysis of subgroups and interactions, sensitivity analysis |  |  |  |  |  |  |  |  |  |  |  |  |  |  |  |  |  |  |  |  |  |
| 18 | Key results | Summarize key results with reference to study objectives | **+** | **+** | **+** | **+** | **+** | **+** | **+** | **+** | **+** | **+** | **+** | **+** | **+** | **+** | **+** | **+** | **+** | **+** | **+** | **+** | **+** |
| 19 | Limitations | Discuss limitations of the study, taking into account sources of potential bias or imprecision. Discuss both direction and magnitude of any potential bias | **+** |  |  |  |  |  | **+** |  |  |  |  | **+** | **+** | **+** | **+** | **+** |  | **+** | **+** | **+** | **+** |
| 20 | Interpretation | Give a cautious overall interpretation of results considering objectives, limitations, multiplicity of analyses, results from similar studies and other relevant evidence | **+** | **+** | **+** | **+** | **+** | **+** | **+** | **+** | **+** | **+** | **+** | **+** | **+** | **+** | **+** | **+** | **+** | **+** | **+** | **+** | **+** |
| 21 | Generalizability | Discuss the generalizability (external validity) of the study results |  |  |  |  |  |  |  |  | **+** |  |  | **+** | **+** |  |  |  |  |  | **+** | **+** |  |
| 22 | Funding | Give the source of funding and the role of the funders of the present study and, if applicable, for the original study on which the present article is based. |  |  |  |  |  |  | **+** |  |  | **+** | **+** | **+** |  | **+** |  |  | **+** |  |  | **+** |  |

Note: The study by Baker at al. has not been assessed for methodological quality, as the data included in the review comes from a conference abstract and the full study is not yet available at the time of writing.
