# Supplementary material for: Shared Sanitation versus Individual Household Latrines: A Systematic Review of Health Outcomes
Source: PLoS One. 2014 Apr 17;9(4):e93300. doi: 10.1371/journal.pone.0093300 (PMC3990518; doi:10.1371/journal.pone.0093300)
Supplement: Table S4 — Extracted data. (DOCX) [file pone.0093300.s008.docx]

**Table S4. Extracted data**

| **PUBLISHED LITERATURE** | | | | | | | | | |
| --- | --- | --- | --- | --- | --- | --- | --- | --- | --- |
| **Reference** | **Country (location)** | **Year** | **Design** | **Population** | **Sampling/Analysis** | **Outcome of interest** | **Measure** | **Study quality** | **Notes** |
| Brooks | Kenya - Rural | 2003 | Case-control study | Patients presenting at clinic with bloody diarhoea | Multivariate logistic regression  2 controls for each case, matched on age and sex | Incidence of bloody diarrhoea | Multivariate analysis showed that  ‘allowing other families to use the compound latrine’ increased the risk of sporadic bloody diarrhoea. [Matched OR 2·76 ( 95% CI 1·26–6·06)]  Specifically for dysentery, the exposure: ‘Allowed other families to use their latrine’ occurred in 34/94 (36%) cases and 32/145 (22%) controls. This provided a Matched OR of 2·40 (CI 1·19- 4·84), p= 0·01 | 16/22  [STROBE]  Due to difficulties locating controls, few were recruited within the same timeframe as cases |  |
| Chakraboty | India - urban | 1983 | Comparative cross sectional | 200 children (<5 years) | 100 children randomly selected from slum, 100 children from multi-story building | Incidence of diarrhea | Average nr of episodes of diarrhea per child during 10 month observation period was 1·6 in slum, and 1·4 in buildings. No statistical methods were performed. | 9/22  [STROBE]  No mention of what random selection method used | Inadequate information to calculate confidence intervals |
| Chandiwana | Zimbabwe-rural | 1989 | Cross sectional study | 1635 farm workers and their families from 15 large scale agricultural communities | Stool sample analysis performed.  Spearman correlations were calculated to investigate relationships between parasitological measurements and the nr of households per latrine in each community | Prevalence and intensity of infection of hookworm and round worm | There were no significant correlations between nr of households per latrine and hookworm prevalence (r=0·7168, t test p<0·1) and with hookworm geometric mean egg count (r=0·7783, t test p<0·1). Similarly, there was no significant correlation between the nr of households per latrine and the roundworm prevalence (r=-0.009, t test p<0.1) | 11/22  [STROBE]  Only 36 latrines in the study area- no clear information on how these 36 were distributed among the 15 communities | No data on whether the nr of households per latrine were counted or calculated as an average |
| Curtale | Egypt- urban | 1998 | Comparative cross sectional | 408 male subjects, aged 8-19, chosen randomly from people aged below 20 and working in a private workshop or commercial activities in the Alexandria governate | Differences calculated using a one-way ANOVA, after log (n+1) transformation of the data.  Bivariate analyses were conducted for the 3 most prevalent parasites to detect significant associations between intensity of infection and exposures | Prevalence and intensity of infection of intestinal helminths | Sharing latrine with other families and the absence of piped water inside the house were associated with a significantly higher intensity of infection for *A. lumbricoides* (p<0·001) and for *T.trichiura* (p<0·05) but not *for S.mansoni* | 12/22  [STROBE] | Results not separated for sharing a latrine and the absence of piped water inside the house.  Efforts have been made to contact the author |
| Ghosh | India- Urban | 1994 | Case control study | 980 rural families with children less than 3 years old living near Calcutta | Descriptive statistics | Diarrhoeal disease | At the end of one year, 570 (58·2%) of 980 families had diarrhoea cases and 410 (42·8%) families had no study children with diarrhoea.  In n=38 or 36·2% (out of n=105 diarrhoeal families) shared a latrine, whereas n=7 or 15·9% (out of n=49) non-diarrhoeal families shared latrines. This is stat sig at p=0·008 (CI not presented in study) | 9/22  [STROBE]  No information on how control families were selected | Additional calculation done:  SD 0·07  95% CI: 0·08-0·32 |
| Golding | Jamaica - Urban | 1994 | Comparative cross sectional | 9919 mothers delivering a singleton in a specified 2 month period and 1847 mothers who delivered a singleton perinatal death in a contiguous 12 month period | Chi-squared tests  Logistic regression | Perinatal death  Antepartum fetal death | Increased risk of perinatal death among women who had to share toilet facilities. This was associated especially with antepartum fetal deaths (p<0·001)  If the toilet was used by people other than family:    Logistic regression analysis  Adjusted OR for predict antepartum fetal death 1·62 [95% CI 1·28,2·03]  Adjusted OR to predict perinatal death using social and environmental factors:  1·41 [95% CI 1·21-1·64] | 14/22  [STROBE] |  |
| Hall | Bangladesh- Urban | 1994 | Comparative cross sectional | Stool samples from 880 residents of an urban slum in Dhaka were collected on 3 occasions over 1 year | Questionnaire  Stool samples  No information on how respondents were selected for questionnaire or stool sample | *Strongyloides stercoralis* infection | Proportion of individuals infected with *S. stercoralis* (at any of the 6-monthly examinations)  Site of children’s defaecation  No.infect. OR CI  Own latrine 18/217 (8·3%) -  Shared latrine 7/157 (4·5%) 0·52(0·22-1·24)  Community lat 35/194 (18·0%) 2·43(1·35-4·38)  Indiscriminate 41/294 (14·7%) 1.79(1·01-3·17)  Site of respondents defecation  No.infect. OR CI  Own lat 17/234 (7.3%) -  Shared lat 18/233(7.7%) 1.07(0.55-2.08)  Comm lat 59/336(17.6%) 2.72 (1.57-4.72)  Indiscrim. 8/71 (11·3%) 1·62 (0·68-3·88) | 13/22  [STROBE] |  |
| Khan | Bangladesh- urban | 1987 | Comparative cross sectional | Inhabitants of two similar per-urban slums of Dhaka | No mention of random selection of study sites  Besides latrine provision, the two study sites were comparable | Diarrhoea incidence and intestinal parasite prevalence (*T. trichiura, E. hystolitica, G. lamblia, S.stercoralis*) | The Kalsi area was provided with 5 communal latrines  In the Tongi area, 78% of the people used communal latrines, 6% used pit latrines and 16% had no definite latrine. In the Kalsi area, 69% used open pit latrines and 31% had no definite latrine.  The rate of diarrhoea, from all causes, did not differ between the Tongi and Kalsi areas | 10/22  [STROBE] |  |
| Kim-Farley | Taiwan- rural | 1984 | Case –control study | Cases reporting through routine reporting channels or through active surveillance system | Study sites chosen because they represented contiguous rural areas with very different attack rates. Log linear modelling and logistic regression analysis | Poliomyelitis | Univariate analysis of differences between case and non-case families in Yun Lin county.  Toilets shared by families, cases had an OR of 4·0 (1·9-8·3) compared to controls. (p value 0·0002) [n=32 cases, n=210 controls]  Univariate analysis of differences between non-case families in Yun Lin and non-case families in Chia Yi counties.  24·3% of families (n=210) in Yun Lin shared latrines vs 15% in Chai Yi (n=200)  OR 1·6 (1·0-2·7) [p=0·0453] | 13/22  [STROBE]    No multivariate data presented | Assuming latrines shared by families was compared to latrines shared by more than just family |
| Mahfouz | Egypt – urban | 1997 | Cross sectional study | Questionnaire from 1324 families  Stool samples from  658 preschool children  below 5 years of age | Questionnaire  Stool samples  Study site was chosen randomly, though method not described  Multivariate analysis  Maximum likelihood estimates of combined OR and their 95%CI adjusted for confounders | Intestinal parasites | Exposure ‘Sharing toilets with other family‘  Multivariate, adjusted Odds ratios  HELMINTHS  1·95(1·38-2·75)  PROTOZOA  1·65(1·06-2·58)  (both at p=0·1) | 13/22  [STROBE] |  |
| Montgomery | Tanzania- rural | 2010 | Case control study | Sub-study population (part of a larger case control)  Children aged 1-5 years.  All households in substudy with sentinel child with clinical signs of active trachoma. Control households were randomly selected from the area. | Questionnaire, blinded to reduce interviewer bias  Logistic regression modelling using generalised estimating equation techniques  630 households were identified, 593 surveys were completed (92 cases 501 controls) for a response rate of 94% | Trachoma  Sanitation sharing practices of households that used latrines | Latrine sharing is practiced by 48·6% of cases and 47·0% of controls.  Of all latrine sharing households, the largest proportion shares a facility with just one neighboring household (20·0% cases, 23·7% controls).  The maximum number of households sharing a latrine was 9. Sharing among 5 or more households is rare (15 instances) and therefore, these were grouped in the category of “four or more households sharing a latrine.”  Results indicate that shared latrines provide as much protection compared with private latrines in regard to risk of trachoma. Adjusted OR= 0·95 (0·55-1·67)  The number of households sharing a latrine does not significantly alter the association | 13/22  [STROBE] |  |
| Munoz | Australia-rural | 1992 | Historical cohort | Hospital admissions for 1961 children from 10 rural aboriginal communities in the Northern Territory | Generalised linear interactive modelling software was used to calculate the nr of admissions per child-year at risk (admission rate) for each community | Hospital admissions | In the factor analysis, communal toilets was of significant (p<0·01) importance. Authors note that these significance levels may be biased, but they indicate the relative importance of each variable for each factor.  Most houses had inside toilets, but some had access only to communal toilets. (ranging from 0-60%)  Although many community characteristics were strongly associated with differences in admission rates between communities, interferences about the causal significance of individual variables cannot be made easily, because at least some of the association will be indirect and non-causal. | 17/22  [STROBE] |  |
| Olusanya | Nigeria - urban | 2010 | Historical  cross-sectional | Women giving birth at an inner-city tertiary maternity hospital- all live births were eligible for enrolment | Cross tabulation  Two-tailed chi square test  Backward stepwise multivariable logistic regression | Preterm birth and low birth weight | Risk Factors for Low Birth weight:  Living in a house with shared sanitation facilities (aOR, 1·27; 95% CI, 0·98–1·65) was retained in the model but had a weak association with low birth weight (P = 0·07).  Risk factors for prematurity  Living in a house with shared sanitation facilities  Prematurity final model,adjusted  aOR (95% CI) = 1·26 (1·07–1·48)  PAR (%) =11·18  Low birth weight final model,  aOR (95% CI) =1·27 (0·98–1·65) (p=0·07)  PAR (%) =12·52 | 18/22  [STROBE] |  |
| Phiri | Malawi – urban and rural | 2001 | cross sectional study | Children aged 3-14 years old residing in either of the two areas of investigations: a rural community and a very densely populated township | Questionnaire and stool samples  ANOVA  Multiple logistic regression  Maximum likelihood estimates obtained  Clear and random sampling frame for areas of study described | Prevalence of Helminth infection (*A. lumbricodes*, *T. trichiura*, *S. stercolralis)* | 73% of urban (n=195) shared a latrine, and 13% in rural (n=13)  Some of the non-significant variables (did not meet the p<0·1 criteria for inclusion in the multivariate analysis model), were sex and age of the child, parental occupation, sharing a latrine, source and storage containers of drinking water and geophagy. | 18/22  [STROBE] | Sharing a latrine was not found to be a statistically significant exposure, thus not included in model |
| Shultz | Kenya- camp setting | 2009 | Cases presenting at IRC camp hospital | Historical case control study | Standardised questionnaires administered to cases and controls  Matched cases to controls by location of residence in the camp and age  Randomised control finding strategy | Watery diarrhoea | UNIVARIATE  N=90 for cases  N=170 for controls  Exposure: Fifteen or more people sharing the same latrine  Cases: 31/52 (60%) Controls:54/112 (48%) MOR 1·5 (0·7, 3·3) P value: 0·33  Exposure: Three or more households sharing same latrine  Cases: 34/51 (67%) controls: 57/111 (51%) MOR 1·9 (0·9, 4·4) P value 0·11  MULTIVARIATE MOR  Exposure: Three or more households sharing same latrine: MOR 2·17 (1·01–4·68) was associated with an increased risk of watery diarrhoea | 16/22  [STROBE] | Camp setting- all that different from very densely populated urban areas? |
| Sobel | Brazil- urban | 2004 | Case control study | Children aged 12-59 months with diarrhoea presenting at hospital | Aged-matched controls  Questionnaire  Multivariate logistic analysis | Acute diarrhoeal disease | Risk factor associated with diarrheal illness in matched pairs of children: sharing toilet with other household MOR 1·48 (1·07-2·04) p=0·02 | 16/22  [STROBE] |  |
| Tshikuka | DRC- Urban | 1995 | Cross- sectional survey | 42 households in each subdivision | Face-to-face interview  Stool sample from single index child randomly selected from each household  Associations between infection and exposure variables were assessed by univariate and multivariate regression procedures for each subdivision independently  MRA.  Randomly selected subdivision | *Ascaris lumbricoides* infection | Multiple regression analysis results  Persons per toilet: Beta= 0·45, coefficient 0·07 (S.E. 0·02). This was significant at p<0·01  Families who regularly defecated in the open had a mean number of 27.0 (S.E. 1·9) person/toilet whereas those who did not had a 18·6 (S.E. 1·4) persons/toilet. According to the authors, persons per toilet can be interpreted either as an index of sanitation or as an index of crowding.  Therefore if there are more persons/toilet, it is more likely that people will (continue to) defecate in the open | 16/22  [STROBE] | It is unclear whether the persons/toilet were counted or calculated as an average |
| Tuttle | Zambia- urban | 1995 | Case control study | Case patient presenting at clinic when interviewers were present | 2 controls for each case matched by age and sex  Multivariate analysis used conditional  logistic regression.  Questionnaire  Stool sample | *Shigella dysenteriae type I* | 64% of cases, versus 46% of controls shared latrines. OR=3·3 (1·1-10·2). p=0·3 | 15/22  [STROBE] | “ It is possible that houses clustered together function in some ways as a single unit, with many forms of close contact occurring among household members” |
| Moshabela | South Africa- rural | 2012 | Case- control | Black African individuals reporting prevalent diarrhoea | Univariate and multivariate logisitic regression.  3 randomly selected controls from same HIV clinic | Diarrhoeal disease | Where data were available, 22/87 (25.3%) of cases and 69/291 controls (23.7%. p=0.763) reported sharing sanitation facilities with other households. An average of 2 households shared sanitation facilities in both cases (range 2-4) and controls (range 2-5). | 19/22 [STROBE] |  |
| Karkey | Nepal- Urban | 2013 | Matched case-control | All febrile patients attending the outpatient or emergency department of Patan Hospital, Kathmandu | Matched univariate analysis, conditional logistic regression. Matched multivariate analysis. | Enteric infection with either *S*. Typhi or *S*. Paratyphi A. | Cases using a household latrine versus those using a community latrine had an adjusted OR of 4.92 (1.2-19.5) for *S.* Parathyphi A.  Cases using a household latrine versus those using a community latrine had an adjusted OR 7.26 (1.4-37.2) for *S*. Typhi.  In total, 92.2% of cases used a household latrine, versus 77.9% of the controls. | 17/22  [STROBE] |  |
| Mahamud | Kenya- refugee camp | 2012 | Matched case-control | Refugees and non-refugees presenting at Kakuma IRC with watery diarrhoea | Bivariate analysis using matched conditional logistic regression. Multivariate conditional logistic regression. | Watery diarrhoea/Cholera | Sharing a communal latrine with neighbouring household was associated with an increased risk of illness (OR, 3.33 (1.34-8.30, p=0.001).  Among the cases, 41% used a communal latrine, versus 23.9% of the controls. | 15/22  [STROBE] |  |

| Appendix V **GREY LITERATURE** | | | | | | | |
| --- | --- | --- | --- | --- | --- | --- | --- |
| Author/  Institution | Year | Title | Country/ Type of document | Conclusions | Quality criteria | Reviewed  Yes/not specified | notesNote Notes |
|  | | | | | | | |
| Baker | 2011 | The risk of moderate and severe diarrhea in children less than 5 years old is increased among families who share a sanitation facility | Various  Conference abstract | Outcome: diarrhoeal disease  Families of case children more commonly used shared sanitation facilities than control families (47·5% vs. 41·2%, mOR = 1·2; 95% CI: 1·1–1·3), overall and in Pakistan (mOR=1·7; 1·4-2·0), Mali (mOR=1·2; 1·1-1·4), India (mOR=1·3; 1·0-1·6), and Kenya (mOR=1·2; 1·0-1·5).  The odds of diarrhoea for shared sanitation were increased two-fold if feces was present (mOR=2·2; 1·6-3·2) than if was absent (mOR=1·2; 1·1-1·3)  While access to unshared sanitation facilities was more common among higher-income households, shared sanitation facilities were consistently more common among case than among control households across all wealth index quintiles.  Our observations indicate that shared sanitation facilities can increase the risk of diarrhea, regardless of the type of facility, and supports their classification as “unimproved”. Increasing access to private sanitation facilities may reduce diarrhea incidence among young children | Inadequate information for proper assessment of study | Abstract presented at ASTMH conference. Final data not yet published. |  |
