# Supplementary material for: Shared Sanitation versus Individual Household Latrines: A Systematic Review of Health Outcomes
Source: PLoS One. 2014 Apr 17;9(4):e93300. doi: 10.1371/journal.pone.0093300 (PMC3990518; doi:10.1371/journal.pone.0093300)
Supplement: Table S5 — Excluded documents. (DOCX) [file pone.0093300.s009.docx]

**Table S5. Excluded documents**

| **Study** | **Reason for exclusion** |
| --- | --- |
| Ademuwagun, 1976 | No results reported on shared sanitation |
| Adewara, 2011 | No results reported on shared sanitation |
| Adewara, 2011 | No health outcomes on shared sanitation |
| Alemayehu, 2005 | No results reported on shared sanitation |
| Amaka, 2003 | No Health outcomes on shared sanitation |
| Amnesty International, 2010 | Reports on shared sanitation, no health outcomes |
| Andereck, 2012 | No health outcomes on shared sanitation |
| Anonymous, 2001 | No results on shared sanitation |
| Aryal, 2012 | No health outcomes on shared sanitation |
| Ashraf, 2011 | No health outcomes in abstract presented at ASTMH 2011 |
| Aubrey, 2009 | No results reported on shared sanitation |
| Awasthi 2003 | No results reported on shared sanitation |
| Ayee, 2003 | No results reported on shared sanitation |
| Baker, 2012 | No health outcomes on shared sanitation |
| Bannerjee, 1988 | No results reported on shared sanitation |
| Bapat, 2003 | No results reported on shared sanitation |
| Barnes, 2004 | No results reported on shared sanitation |
| Bassett, 1992 | No results reported on shared sanitation |
| Bateman, 1995 | No results reported on shared sanitation |
| Bhardwaj,2013 | No health outcomes for shared sanitation users |
| Bility, 2000 | No results reported on shared sanitation |
| Bindeshwar, 2011 | No results reported on shared sanitation |
| Biran, 2011 | Very relevant information on communal sanitation, no health outcomes |
| Briscoe, 1992 | No results reported on shared sanitation |
| Butala, 2010 | No results reported on shared sanitation |
| Cameron, 2010 | No results reported on shared sanitation |
| Candidate nr 100559, 2011 | No health outcomes reported |
| Candidate nr 100788, 2011 | No health outcomes reported |
| Candidate nr 491174, 2010 | No results reported on shared sanitation |
| Caplan, 2010 | No results reported on shared sanitation |
| Carden, 2007 | No results reported on shared sanitation |
| Chaggu, 2004 | No results reported on shared sanitation |
| Chimbari,1992 | No results reported on shared sanitation |
| Chitkara, 1986 | No results reported on shared sanitation |
| Chowdhury, 2010 | Medical description of a cholera case |
| Clemens, 1987 | No results reported on shared sanitation |
| Colin, 2007 | No results reported on shared sanitation |
| Colombatti, 2009 | No results reported on shared sanitation |
| Cotton, 1995 | No results reported on shared sanitation |
| Coulson, 2001 | No results reported on shared sanitation |
| Cuesta, 2007 | Descriptive info on shared sanitation, but no results presented |
| Cumberland, 2005 | No results reported on shared sanitation |
| De, 1957 | No results reported on shared sanitation |
| Devadas, 2002 | No results reported on shared sanitation |
| Devereux, 1994 | No results reported on shared sanitation |
| Duncker, 2000 | No results reported on shared sanitation |
| Dunstan, 1998 | Sanitation report, no results on shared sanitation |
| Eales, 2008 | No results reported on shared sanitation |
| Ekanem, 1994 | No results reported on shared sanitation |
| Elhag, 2013 | No health outcomes on shared sanitation |
| Feachem, 1977 | Book. No results reported on shared sanitation |
| Feachem, 1983 | No results reported on shared sanitation |
| Ferreira, 2000 | Parasitological survey, shared sanitation not assessed |
| Fobil, 2010 | No direct health outcomes. Focuses on environmental contamination |
| Garbossa, 2013 | No health outcomes on shared sanitation |
| Gichuri, 1999 | No results reported on shared sanitation |
| Govender, 2010 | No results reported on shared sanitation |
| Gracey, 1997 | No results reported on shared sanitation |
| Grimason, 2000 | No health outcomes |
| Gubler, 1989 | No results reported on shared sanitation |
| Gupta, 1996 | Descriptive study of cholera cases |
| Hare, 1938 | Unobtainable- contacted publisher |
| Hare,1940 | Unobtainable- contacted publisher |
| Heeb, 2003 | No results reported on shared sanitation |
| Heng, 2012 | Review on sanitation, no specific information on shared sanitation |
| Hobson, 2000 | Discusses the design of a communal sanitation project. No results |
| Home Office, 2008 | Report of public sanitation in the UK |
| Hoque, 1994 | No results reported on shared sanitation |
| Hunt, 2001 | No results reported on shared sanitation |
| Hutton,2011 | Review on sanitation, economic assessment |
| Illing, 2009 | No results reported on shared sanitation |
| Jha, 2003 | Mentions Sulabh latrines. No data collected on shared sanitation |
| JMP, 2010 | No Health outcomes on shared sanitation |
| JMP, 2012 | No Health outcomes on shared sanitation |
| JMP, 2013 | No Health outcomes on shared sanitation |
| Jones, 2005 | No results reported on shared sanitation |
| Jones, 2008 | No results reported on shared sanitation |
| Joshi, 2011 | No results on shared sanitation |
| Joshi, 2012 | No health outcomes on shared sanitation |
| Kamundi, 2008 | No results reported on shared sanitation |
| Karanja, 2000 | Unobtainable |
| Karim, 2003 | Unobtainable |
| Kavita, 2013 | No health outcomes on shared sanitation |
| Kuria, 2003 | No results reported on shared sanitation |
| Kuria, 2005 | No results reported on shared sanitation |
| Kwagala, 1999 | Provides some public opinion on shared sanitation, but no results |
| Lagardien, 2005 | No results reported on shared sanitation |
| Lal, 1996 | No results on shared sanitation |
| Lunch, 1983 | No results reported on shared sanitation |
| Mahon, 2010 | No results reported on shared sanitation |
| Mara, 2005 | No results reported on shared sanitation |
| Matthys, 2011 | No health outcomes for shared sanitation users |
| Mayumbelo, 2008 | No results reported on shared sanitation |
| Mazeau, 2010 | No specific results reported on shared sanitation |
| Mbere, 1980 | No results reported on shared sanitation |
| McCann, 2001 | No results reported on shared sanitation |
| McCommon, 1998 | No results reported on shared sanitation |
| McElligott, 2013 | No health outcomes on shared sanitation |
| Molotch, 2010 | Book discusses public latrines from a sociological point of view |
| Moseti, 2009 | No results reported on shared sanitation |
| Mukherjee, 2012 | Reports on shared sanitation, no health outcomes |
| Mukhopadhyay,2008 | No results reported on shared sanitation |
| Mushtaq, 2008 | No results associated with shared sanitation |
| Naidoo, 2007 | No results reported on shared sanitation |
| Naranjo, 2010 | No results reported on shared sanitation |
| Ndugwa, 2008 | No results reported on shared sanitation |
| Neelin, 2011 | No health outcomes on shared sanitation |
| Ngugi, 1992 | Discusses public latrines, not shared sanitation used from a home |
| Ngui, 2011 | No health outcomes on shared sanitation |
| NgyuenViet Anh, 2012 | Guiding policy document on sanitation, economic assessment |
| Nhapi, 2005 | No results reported on shared sanitation |
| Nock, 2002 | Shared sanitation in public institutions, not domestic setting |
| Obika, 2003 | No results reported on shared sanitation |
| Oduro-Kwarteng, 2009 | Reports on shared sanitation, no health outcomes |
| Okiro, 2007 | No results reported on shared sanitation |
| Omishakin, 1986 | Discusses public opinion on shared sanitation, but no data is provided |
| Opisa, 2012 | No health outcomes on shared sanitation |
| Pardeshi, 2009 | No results reported on shared sanitation |
| Parett, 1983 | No results reported on shared sanitation |
| Patel, 2013 | No health outcomes on shared sanitation |
| Pickford, 1990 | Opinion piece, no data collected on shared sanitation |
| Politzer, 2012 | No results reported on shared sanitation |
| Poti, 1963 | Unobtainable-contacted publisher |
| Pratt, 1991 | No results reported on shared sanitation |
| Putrali, 1980 | Pilot study. No results associated with shared sanitation |
| Rashid, 2009 | Policy piece. No results associated with shared sanitation |
| Reuter, 2003 | No results reported on shared sanitation |
| Rheingans, 2006 | No results reported on shared sanitation |
| Roderiguez, 2011 | Review on sanitation, policy document |
| Roma, 2010 | No results reported on shared sanitation |
| Root, 2001 | No results reported on shared sanitation |
| Rosensweig, 1996 | No results reported on shared sanitation |
| Saha, 2012 | No health outcomes on shared sanitation |
| Saywell, 1996 | No results reported on shared sanitation |
| Scott, 1999 | No results reported on shared sanitation |
| Seager, 1994 | No results reported on shared sanitation |
| Sharma, 2011 | No health outcomes on shared sanitation |
| Shobha, 2013 | No health outcomes on shared sanitation |
| Sikwibele, 1996 | Descriptive info, opinions on shared sanitation, no results reported |
| Silva, 2011 | No health outcomes on shared sanitation |
| Simpson-Hebert,1998 | Review on sanitation, not specifically shared sanitation |
| Siu, 2008 | Discusses public sanitation for visually impaired in developed countries |
| Sobrinho, 1995 | Shared sanitation in public institutions, not in domestic setting |
| Sridhar, 1999 | No results reported on shared sanitation |
| Stanton, 1985 | Descriptive info on shared sanitation, but no results |
| Strudwick, 1962 | No results reported on shared sanitation |
| Sulabh International, 2012 | No health outcomes |
| Sur, 2007 | No results reported on shared sanitation |
| Swami, 2004 | No results reported on shared sanitation |
| Tettey-Lowor, 2009 | No results reported on shared sanitation |
| Thapa Magar, 2011 | No health outcomes on shared sanitation |
| Thomas, 1999 | No results reported on shared sanitation |
| Tiimub, 2009 | Information collected on shared sanitation, but no results provided |
| Tumwebaze, 2013 | No health outcomes on shared sanitation |
| Udayani, 1999 | No results reported on shared sanitation |
| Van Ryneveld, 2003 | No results reported on shared sanitation |
| Van Wijk, 1997 | No results reported on shared sanitation |
| Von Munch, 2005 | Information collected on shared sanitation, but no results provided |
| Water Research Commission, 1993 | Sanitation report, no specific results on shared sanitation |
| WaterAid, 2007 | Sanitation report, no specific results on shared sanitation |
| WaterAid, 2008 | Sanitation report, no specific results on shared sanitation |
| WaterAid, 2011 | Sanitation report, no specific results on shared sanitation |
| WaterAida, 2008 | Sanitation report, no specific results on shared sanitation |
| WEDC, 1986 | Sanitation conference proceedings, no results on shared sanitation |
| Wegelin-Schuringa, 1997 | Review on sanitation, not specifically shared sanitation |
| Westaway, 1998 | Sanitation report, no specific results on shared sanitation |
| Whittington, 1993 | No results reported on shared sanitation |
| Worrell, 2012 | No health outcomes in abstract presented at ASTMH 2011 |
| WRC, 1998 | Sanitation report, no specific results on shared sanitation |
| WRC, 1998 | No Health outcomes on shared sanitation |
| WSP, 2001 | Guiding policy document on sanitation, economic assessment |
| WSP, 2006 | Guiding policy document on sanitation, economic assessment |
| WSP, 2010 | Guiding policy document on sanitation, economic assessment |
| WSP, 2011 | Guiding policy document on sanitation, economic assessment |
| WSP, 2012 | Guiding policy document on sanitation, economic assessment |
| WSPa, 2010 | Guiding policy document on sanitation, economic assessment |
| WSPb, 2010 | Guiding policy document on sanitation, economic assessment |
| WSPc, 2010 | Guiding policy document on sanitation, economic assessment |
| WSUP, 2010 | Sanitation report, no specific results on shared sanitation |
| WSUP, 2011 | Sanitation report, no specific results on shared sanitation |
